# Varied Pathways of Infant Gut-Associated *Bifidobacterium* to Assimilate Human Milk Oligosaccharides: Prevalence of the Gene Set and Its Correlation with Bifidobacteria-Rich Microbiota Formation

**DOI:** 10.3390/nu12010071

**Published:** 2019-12-26

**Authors:** Mikiyasu Sakanaka, Aina Gotoh, Keisuke Yoshida, Toshitaka Odamaki, Hiroka Koguchi, Jin-zhong Xiao, Motomitsu Kitaoka, Takane Katayama

**Affiliations:** 1 National Food Institute, Technical University of Denmark, Kemitorvet, DK-2800 Kgs. Lyngby, Denmark; miksak@dtu.dk; 2 Research Institute for Bioresources and Biotechnology, Ishikawa Prefectural University, Nonoichi, Ishikawa 921-8836, Japan; 3 Graduate School of Biostudies, Kyoto University, Kyoto 606-8502, Japan; 4 Next Generation Science Institute, Morinaga Milk Industry Co., Ltd., Zama, Kanagawa 252-8583, Japan; keisuke-yoshida826@morinagamilk.co.jp (K.Y.); t-odamak@morinagamilk.co.jp (T.O.); j_xiao@morinagamilk.co.jp (J.-z.X.); 5 Department of Biotechnology and Bioengineering, Technical University of Denmark, Søltofts Plads, DK-2800 Kgs. Lyngby, Denmark; hirosa@dtu.dk; 6 The Faculty of Agriculture, Niigata University, Niigata 950-2181, Japan; mkitaoka@agr.niigata-u.ac.jp

**Keywords:** *Bifidobacterium*, breast-feeding, human milk oligosaccharides, infant, microbiota

## Abstract

The infant’s gut microbiome is generally rich in the *Bifidobacterium* genus. The mother’s milk contains natural prebiotics, called human milk oligosaccharides (HMOs), as the third most abundant solid component after lactose and lipids, and of the different gut microbes, infant gut-associated bifidobacteria are the most efficient in assimilating HMOs. Indeed, the fecal concentration of HMOs was found to be negatively correlated with the fecal abundance of *Bifidobacterium* in infants. Given these results, two HMO molecules, 2′-fucosyllactose and lacto-*N*-*neo*tetraose, have recently been industrialized to fortify formula milk. As of now, however, our knowledge about the HMO consumption pathways in infant gut-associated bifidobacteria is still incomplete. The recent studies indicate that HMO assimilation abilities significantly vary among different *Bifidobacterium* species and strains. Therefore, to truly maximize the effects of prebiotic and probiotic supplementation in commercialized formula, we need to understand HMO consumption behaviors of bifidobacteria in more detail. In this review, we summarized how different *Bifidobacterium* species/strains are equipped with varied gene sets required for HMO assimilation. We then examined the correlation between the abundance of the HMO-related genes and bifidobacteria-rich microbiota formation in the infant gut through data mining analysis of a deposited fecal microbiome shotgun sequencing dataset. Finally, we shortly described future perspectives on HMO-related studies.

## 1. Introduction

Bifidobacteria are gram-positive anaerobes that inhabit animal digestive tracts and dairy products, with 78 species and 10 subspecies taxonomically identified to date (a total of 84 taxa) [1,2,3,4,5,6,7,8,9,10,11]. More than ten (sub)species were isolated from human stools, and among them, six (sub)species are known to be frequent colonizers of infant guts (described later) [1,12,13,14]. *Bifidobacterium* is generally the most abundant taxon of the infant gut microbiota (up to 90%), and the richness is associated with various beneficial effects on infant health, that include folate production in the intestines [15], increased immune responses to vaccinations [16], and prevention or reduction of allergic diseases [17]. Tight adherence pili produced by bifidobacteria are known to stimulate colonic epithelial proliferation, and thereby may influence the maturation of the neonatal gut [18]. The healthy bifidobacteria-rich gut microbiota continues during breast-feeding, but the relative abundances rapidly decrease after weaning [12,19]. During this transition, the compositional change occurs at the species level [20]. The species-level change has also been mentioned in elderly individuals (centenarians) [19,20].

*Bifidobacterium* spp. was isolated by Henry Tissier at the Pasteur Institute in 1899 as a bacterium that is dominant in the stools of breast-fed infants but is scarce in formula-fed infant stools. Fifty years later, a research group found that human milk, but not cow’s milk, efficiently stimulated the growth of the bacterium [21]. In a subsequent study, they purified the bifidogenic compound and reported that it is a mixture of oligosaccharides (human milk oligosaccharides: HMOs) composed of l-fucose (Fuc), galactose (Gal), glucose (Glc), and *N*-acetylglucosamine (GlcNAc) [22], which is the third most abundant solid component (10–20 g/L) contained in human milk [23,24,25]. Their report was, however, later disputed as a phenotype that was specific only to the bacterium they used in their growth assay [26], since then, to our knowledge, there had been no reports that link bifidobacteria and HMOs until recently.

In 2004 and 2005, our group isolated the genes for 1,2-α-l-fucosidase and galacto-*N*-biose (GNB)/lacto-*N*-biose I (LNB) phosphorylase from *Bifidobacterium bifidum* and *Bifidobacterium longum* subsp. *longum* (*B. longum*), respectively [27,28]. The activity of these enzymes on host-derived glycans, especially HMOs, as well as the limited occurrence of homologs in gut microbial genomes, with the exception of bifidobacterial genomes, made us reconsider the once dismissed link between bifidobacteria and HMOs. An interesting coincidence is that David A. Mills’s group at the University of California, Davis was also aware of the bifidobacterial capability to assimilate HMOs at that time [29]. His group addressed this issue through genomic sequence analysis of *B. longum* subsp. *infantis* (*B. infantis*) and found that the organism is equipped with a gene set responsible for HMO assimilation [30]. Since then, various researchers have tried to elucidate the mechanisms of how bifidobacteria assimilate HMOs, and of how HMO consumption is related to the gut microbiota formation in breast-fed infant guts [12,31,32,33,34,35,36,37]. Currently, breast-feeding is considered to be one of the most efficient strategies to shape healthy gut microbiotas during infancy [14,38].

In the present review, we first summarize characteristics and physiological roles of the enzymes and transporters responsible for HMO assimilation by bifidobacteria, and then analyze the prevalence and conservation of the gene set among different *Bifidobacterium* species and strains. Publically-available genomes (565) of *Bifidobacterium* residing in infant guts (*B. bifidum*, *Bifidobacterium breve*, *Bifidobacterium kashiwanohense*, *B. infantis*, *B. longum*, and *Bifidobacterium pseudocatenulatum*), adult guts or mouths (*Bifidobacterium adolescentis*, *Bifidobacterium angulatum*, *Bifidobacterium catenulatum*, *Bifidobacterium dentium*, and *Bifidobacterium gallicum*), and non-human animal intestines and dairy products (*Bifidobacterium animalis* subsp. *animalis* (*B. animalis*), *B. animalis* subsp. *lactis* (*B. lactis*), *Bifidobacterium pseudolongum* subsp. *globosum* (*B. globosum*), *B. pseudolongum* subsp. *pseudolongum* (*B. pseudolongum*), and *Bifidobacterium thermophilum*) were used for comparison. Furthermore, we examined the correlation between each gene and the formation of bifidobacteria-rich microbiotas in the infant guts through data mining analysis of deposited fecal microbiome shotgun sequencing data. The results are then discussed based on the different HMO utilization strategies adopted by the respective infant gut-associated bifidobacteria. This review reveals how specific *Bifidobacterium* species/strains have adapted to HMOs and shows how specific bifidobacterial gene sets for HMO assimilation are associated with infant gut microbiota formation.

## 2. Materials and Methods

### 2.1. Homolog Search

The prevalence of HMO and galactooligosaccharide (GOS) utilization genes in the genomes of sixteen *Bifidobacterium* (sub)species was examined by tblastn analysis (BLAST+ v2.9.0) with the following criteria: identity ≥ 70%, query coverage ≥ 60%, and e value < 1 × 10^−50^ (Supplementary Figure S1A). Query amino acid sequences were obtained from GenBank under the accession numbers listed in Table 1 (for HMO-related genes) and from LC015362.1, LC333765.1, AP010888.1, and LC333766.1 (for GOS-related genes). The LnpA, Bga42A, Blon_0459, and Blon_0732 sequences were used as representative queries for the search of GNB/LNB phosphorylase (LnpA, LnpA1, LnpA2, and LnbP), lacto-*N*-tetraose (LNT) β-1,3-galactosidase (Bga42A and LntA), and β-*N*-acetylglucosaminidases (Blon_0459 and NahA; Blon_0732 and BLIJ_1391), respectively. With respect to the solute-binding proteins (SBPs) of GNB/LNB transporter, both GltA (AP010888.1) and BBBR_RS08090 (LC333766.1) were used as search queries (see Section 3.2.1.). A local database was created by retrieving the coding region sequences (CDS) from 565 *Bifidobacterium* genomes deposited in the NCBI reference sequence database, available on 21 June 2019 (https://www.ncbi.nlm.nih.gov/). The accession numbers are listed in Supplementary Table S1. The prevalence (%) of the genes in each (sub)species was determined by dividing the number of the retrieved genes with the above identity criteria by the number of the genomes examined.

### 2.2. Metagenomic Data Mining Analysis

The occurrence of HMO and GOS utilization genes (identity ≥70%, read coverage ≥60%) in infant stool DNA was examined by using the tblastn algorithm (BLAST+ v2.6.0) against the fecal microbiome shotgun sequencing dataset (MG-RAST version 4.0.3 (accession number “qiime:621”); http://metagenomics.anl.gov/) [39] (Supplementary Figure S1B). The quality-filtered reads were obtained as described previously [40], and the resulting shotgun sequencing data of 117,492 ± 55,056 reads/sample (means ± standard deviations) for 34 breast-fed and 27 formula-fed infants (≤1 year old), who resided in USA (*n* = 37), Malawi (*n* = 14) and Venezuela (*n* = 10), were used as the local database. The amino acid sequences used for queries were the same as described above. LnbY was omitted from the analysis due to its short length. The relative abundance (%) of each homolog gene in the fecal DNAs was calculated by dividing the hit read counts with the above identity criteria by total read counts. When redundant mappings were detected, the reads with the highest tblastn scores were chosen to count. If multiple reads had equally high scores, the count value was divided by the number of reads with the same score (e.g., count value = 0.5 for mappings with two reads with equally high scores (1/2); count value = 0.25 for mappings with four reads with equally high scores (1/4)). The number of the reads with multiple equally high tblastn scores was six in total. The abundance (%) of the genus *Bifidobacterium* was obtained from MG-RAST under the same accession number as above. 

## 3. HMO Degradation Machinery

### 3.1. Glycosidases and Phosphorylase

The core structures of HMOs consist of Gal, Glc, and GlcNAc, and the cores are frequently decorated with Fuc and *N*-acetylneuraminic acid (Neu5Ac) [23,24]. Fucosylated HMOs are assumed to account for ≈70% of the total HMOs by weight, while sialylated HMOs are ≈10–20% [25]. HMO mixture comprises more than 150 oligosaccharide structures, and among them, 2′-fucosyllactose (2′-FL) (≈25%), LNT (≈10%), lacto-*N*-fucopentaose (LNFP) I (≈10%), LNFP II (≈10%), and lacto-*N*-difucohexaose (LNDFH) I (≈10%) are known to be major structures in secretor’s milk (Supplementary Figure S2; see Section 6 for secretors/non-secretors) [23,25]. These complex oligosaccharides must be decomposed to drive the fructose 6-phosphate phosphoketolase-dependent glycolytic pathway (bifid shunt) for catabolism. To this end, *Bifidobacterium* has evolved several glycosidases and a phosphorylase with high specificities for host-derived glycans (HMOs and mucin *O*-glycans).

#### 3.1.1. Fucosidase

Two-types of fucosidases are necessary to hydrolyze fucosidic linkages found in HMOs: 1,2-α-l-fucosidase and 1,3-1,4-α-l-fucosidase. 1,2-α-l-Fucosidase (EC 3.2.1.63) is classified into the glycoside hydrolase family 95 (GH95) [27,55], while 1,3-1,4-α-l-fucosidase (EC 3.2.1.51) belongs to the GH29 subfamily B [56]. GH95 α-l-fucosidase has a strict recognition for H-antigen disaccharide structure (Fucα1-2Gal-*O*-R) in the catalytic pocket [57], and therefore, it is highly active on 2′-FL and LNFP I [27]. The enzyme also acts on lactodifucotetraose (LDFT) and LNDFH I with moderate activity [58], and surprisingly, on 3-fucosyllactose (3-FL) to a limited extent (Supplementary Figure S2) [27]. The activity on 3-FL can be explained by the structural mimicry of the α-anomer of 3-FL to H-antigen structure (Supplementary Figure S3). GH29 subfamily B α-l-fucosidase is unique in that it requires a branched Gal residue for the hydrolysis of fucosidic linkage. The enzyme was shown to adopt the induced-fit catalytic mechanism that is triggered by the binding of Gal residue to the active site. Interestingly, the steric position of Fuc residue bound to GlcNAc towards Gal residue is the same between LNFP II (Lewis a trisaccharide) and LNFP III (Lewis x trisaccharide) (Supplementary Figures S2 and S3), which indicates that the enzyme recognizes identical sugar motifs to exert its activity.

#### 3.1.2. Sialidase

Sialidases (EC 3.2.1.18) from bifidobacteria, which belong to GH33, act on both α-2,3 and α-2,6 linkages found in sialylated HMOs (Supplementary Figure S2), whereas the enzyme from *B. bifidum* (SiaBb2) shows a preference for the α-2,3 linkage over the α-2,6 linkage [42]; the enzyme from *B. infantis* (NanH2) hydrolyzes both linkages almost equally [50]. SiaBb2 has been shown to liberate Neu5Ac from mucin glycoproteins [42,59]. Binding of the catalytic domain of SiaBb2 to blood group A substance (GalNAcα1-3(Fucα1-2)Gal) is noteworthy [59].

#### 3.1.3. Lacto-N-Biosidase

Lacto-*N*-biosidase (EC 3.2.1.140) hydrolyzes LNT to produce LNB and lactose (Lac) (Supplementary Figure S2). The enzyme from *B. bifidum* (LnbB) is classified as GH20 [43], while the enzyme from *B. longum* (LnbX) is categorized into GH136 [60]. GH136 enzymes require a designated chaperon (LnbY) for its proper folding. LnbB and LnbX are highly specific for LNT hydrolysis. However, LnbX additionally accepts LNFP I and α-2,3-sialyl LNT (LSTa), although the activity is considerably low [44]. The different activity was also mentioned for two enzymes using the sugar chains of gangliosides and globosides [61]. Disruption of *lnbX* gene caused severe growth retardation on LNT in *B. longum* [60]. Inactivation of *lnbB* in *B. bifidum* has not been attained due to the difficulty of gene manipulation in this species. It is interesting to note that *B. bifidum* and *B. longum* have evolved two completely different genes to catabolize the same substrate, LNT (the most abundant core structure of HMOs), with almost equal kinetic parameters, even though they belong to the same genus and share the same environmental niche.

#### 3.1.4. β-1,4-Galactosidase

Lac and the type-2 chain (Galβ1-4GlcNAc-*O-*R) found in HMOs are susceptible targets of β-1,4-galactosidases (EC 3.2.1.23). BbgIII from *B. bifidum* and LacZ2 and LacZ6 from *B. breve* belong to GH2 [45,48]. Inactivation of the *lacZ2* gene reduced the growth ability of the strain on Lac, while disruption of *lacZ6* gene had no effect on Lac assimilation ability [48]. Neither *lacZ2* nor *lacZ6* knockout affected the growth of *B. breve* on lacto-*N*-*neo*tetraose (LN*n*T) (Supplementary Figure S2) [48].

#### 3.1.5. LNT β-1,3-Galactosidase

*Exo*-glycosidases that act on β-(1→3)-linked Gal are rarely found in nature, and GH2 β-galactosidases from *Bifidobacterium* (see Section 3.1.4) were found to be inactive on type-1 chains (Galβ1-3GlcNAc-*O-*R) [51]. Through a search of the bifidobacterial genomes, a GH42 β-galactosidase (EC 3.2.1.23) that specifically hydrolyzes LNT into Gal and lacto-*N*-triose II (LNTri II) was found (Supplementary Figure S2) [51]. The enzyme, termed LNT β-1,3-galactosidase, shows the highest activity towards LNT, followed by Lac, LNB, and LN*n*T. The hydrolytic ability of this enzyme for both type-1 and type-2 chains is in sharp contrast to the above-mentioned β-1,4-galactosidases that is essentially inert on type-1 chains. The structural basis for the dual activity of LNT β-1,3-galactosidase remains to be elucidated. Disruption of the gene (*lntA*) eliminated the growth ability of *B. breve* on LNT [48].

#### 3.1.6. β-N-Acetylglucosaminidase

Several GH20 β-*N*-acetylglucosaminidases (EC 3.2.1.52) have been isolated from bifidobacteria. The enzyme from *B. bifidum* (BbhI) and three enzymes from *B. infantis* (Blon_0459, Blon_0732, and Blon_2355) are highly active on LNTri II (Supplementary Figure S2) [45,53]. Blon_0459 and Blon_0732 were also shown to act on lacto-*N*-hexaose (LNH; Galβ1-3GlcNAcβ1-3(Galβ1-4GlcNAcβ1-6) Galβ1-4Glc) after removing the terminal β-1,3 linked Gal residue. Blon_0459 and Blon_0732 also displayed low and high activities for β-1,6 linked GlcNAc residues in LNH after cleaving the terminal β-1,4 linked Gal residue, respectively. β-*N*-Acetylglucosaminidase from *B. longum* (BLLJ_1391, a homolog of Blon_0732) was found to act on LNTri II and *N*,*N’*-diacetylchitobiose equally [52]. *B. breve* UCC2003 possesses one GH20 enzyme (Bbr_1556, a Blon_0459 homolog) in its genome, and disruption of the gene (*nahA*) impaired the ability of the strain to grow on LNT and LN*n*T [48].

#### 3.1.7. GNB/LNB Phosphorylase

GNB/LNB phosphorylase (EC 2.4.1.211) is an intracellular enzyme that reversibly phosphorolyzes GNB/LNB to generate α-galactosyl 1-phosphate (Gal1*P*) and GalNAc/GlcNAc. This enzyme is categorized in GH112 [55]. Compared to hydrolysis of the same substrate, phosphorolysis conserves ATP consumption during the metabolism of GNB/LNB in cells, because the resultant Gal1*P* is converted into fructose 6-phosphate without consuming ATP (Leloir pathway requires ATP to produce Gal1*P* from the liberated Gal) [28]. Insertional mutation into the GNB/LNB phosphorylase gene (*lnbP*) caused the impaired growth of *B. breve* on LNB [48].

### 3.2. Transporters

**Figure 1:**
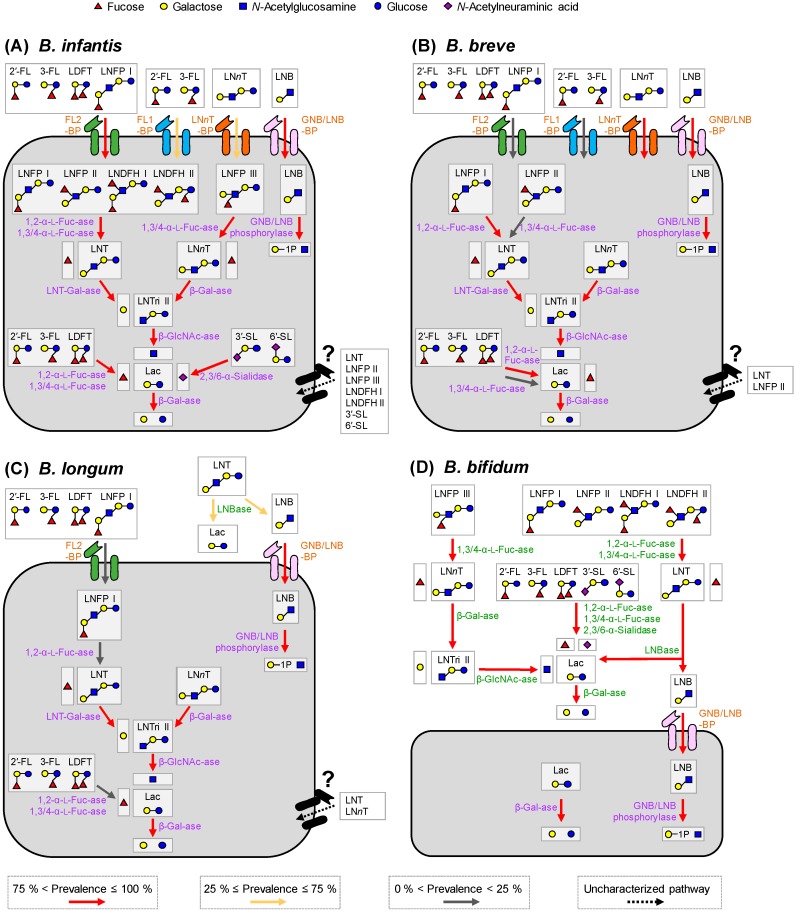
HMO utilization pathways in the four major infant-gut associated *Bifidobacterium* species. (**A**–**D**) Degradation pathways for the representative 12 HMO molecules in *B. infantis* (**A**), *B. breve* (**B**), *B. longum* (**C**), and *B. bifidum* (**D**) are shown. The pathways in *B. kashiwanohense* and *B. pseudocatenulatum* are shown in Supplementary Figure S4. The arrows with different colors indicate the prevalence of respective homolog genes in each species (see Figure 2). Red: >75%; yellow, 25−75%; and gray: <25%. The uncharacterized degradation pathways are indicated by dotted black arrows. The extracellular enzymes and transporter homologs are shown in green and brown letters, respectively, while intracellular enzymes are in purple letters. Mono and di-saccharides liberated outside the cell membranes can be shared among gut bacteria, especially among bifidobacteria.

As described later, bifidobacteria have evolved two different strategies for assimilating HMOs. One is transporter-dependent (intracellular digestion strategy), and the other is extracellular glycosidase-dependent (extracellular digestion strategy) (Table 1, Figure 1, Supplementary Figure S4). The presence of a transporter-dependent HMO consumer was first revealed by the genomic analysis of *B. infantis* ATCC 15697^T^ by David A. Mills’s group [30]. The strain possesses the so-called HMO cluster that is comprised of several intracellular HMO-related glycosidases as well as SBPs for ATP-binding cassette-type (ABC) transporters. Seven SBPs are in the cluster, while the genome encodes for an additional thirteen SBPs in different loci. Mills’s group subsequently analyzed the specificity of the SBPs using glycan array [62], which enhanced our understanding of HMO catabolism in bifidobacteria with the intracellular digestion strategy. However, studies on HMO transporters are still behind compared to the aforementioned glycosidases involved in the decomposition of HMOs. Indeed, only three transporters for LNB, FL, and LN*n*T have been physiologically characterized.

#### 3.2.1. GNB/LNB Transporter

The GNB/LNB transporter was first isolated and identified to be responsible for the uptake of LNB that is extracellularly liberated from LNT by lacto-*N*-biosidase (LnbB from *B. bifidum* and LnbX from *B. longum*) [43,44] and for the uptake of GNB that is liberated from mucin *O*-glycans by endo-α-*N*-acetylgalactosaminidase [63]. Introduction of a plasmid carrying the transporter genes (*gltABC*) into *B. longum* increased the accumulation level of ^14^C-labeled LNB inside the cells, compared to the control strain carrying an empty vector [31,64]. The SBP of the transporter (GltA, or GNB/LNB-BP) from *B. longum* specifically binds LNB and GNB with *K*_d_ values of 87 and 10 nM, respectively [46]. The recent study also revealed that GltA of *B. breve* (78% identity to GltA of *B. longum*) can bind 3′-galactosyllactose (GOS) with *K*_d_ values of 2 × 10^4^ nM. Note that eight amino acid residues involved in the binding of LNB are completely conserved between all GltA homologs mentioned in this study, except for the asparagine residue at the position of 206 of *B. longum* GltA [46]. 

#### 3.2.2. FL Transporter

The FL transporter of *Bifidobacterium* was first identified by Yakult Co. Ltd.’s group and Mills’s group, independently [12,65]. They reported that the transporter has an ability to import 2′-FL, 3-FL, LDFT, and LNFP I. *B. infantis* ATCC 15697^T^ possesses two paralogous FL transporters with their SBPs sharing 60% identity. Biochemical and structural analyses, combined with genetic analysis, unequivocally revealed that the functions of the two transporters are overlapping but distinct [40]. One (designated FL transporter-1 [SBP name: FL1-BP]) imports 2′-FL and 3-FL only, while the other (FL transporter-2 (SBP name: FL2-BP)) is capable of internalizing LDFT and LNFP I in addition to 2′-FL and 3-FL. The wild type strain of *B. infantis* outcompeted the transporter knockout strain to grow on a purified HMO mixture [40]. While FL1-BP binds preferentially to 2′-FL (the binding to 3-FL was not observed by isothermal calorimetry analysis under the tested conditions), FL2-BP binds to 2′-FL and 3-FL with the *K*_d_ values of 5.4 and 6.0 μM, respectively. This intriguing dual specificity has been rationalized by X-ray crystal structure analysis of the ligand-complexed structures [40].

#### 3.2.3. LN*n*T Transporter

LN*n*T transporter has been genetically identified in *B. breve* UCC2003 by van Sinderen’s group [48]. James et al. [48] conducted a series of targeted gene knockouts based on the results obtained in the transcriptome analysis of cells grown on HMO-related compounds including LN*n*T and then monitored the growth of the mutants in media supplemented with several oligosaccharides. Consequently, *nahS* (LN*n*T-BP) was found to be the SBP responsible for the uptake of LN*n*T.

## 4. HMO Utilization Strategy

### 4.1. In Vitro HMO Consumption by Bifidobacteria

HMO consumption behavior of bifidobacteria has been examined using purified HMO mixtures, or each available HMO molecule as a sole carbon source [12,32,48,58,65,66,67,68,69,70,71]. *B. bifidum* and *B. infantis* efficiently assimilate almost all structures of HMOs (including sialylated HMOs) and LNB (Supplementary Figure S2), and the assimilation ability is highly conserved among the strains belonging to these (sub)species [32,58,66,67,69,70]. *B. infantis* JCM 1260, which shows diminished growth on HMOs, has an incomplete HMO cluster in the genome [71]. In contrast to the above two species, *B. breve* and *B. longum* show a limited capability to catabolize HMOs. The majority of *B. breve* strains are reported to assimilate LNT, LN*n*T, and LNB only [32,48,58,67,68,70]. The accessibility of HMOs for *B. longum* is essentially limited to LNT and LNB [32,58,65,67]. Utilization of the other HMOs, such as 2′-FL, 3-FL, LDFT, and LNFP I/II/III, has been reported for only a few strains of *B. breve* and *B. longum* [12,65,68].

HMO assimilation by *B. pseudocatenulatum* and *B. kashiwanohense* has also been recognized. Xiao et al. [67] demonstrated that 33 out of 61 strains of *B. pseudocatenulatum* are able to utilize LNB. Matsuki et al. [12] reported LNT consumption by *B. pseudocatenulatum*. They also mentioned that some strains of this species assimilate 2′-FL, 3-FL, and LDFT. *B. kashiwanohense* was shown to be a good consumer of 2′-FL and 3-FL, and the ability is well conserved in this species [70]. The ability of *B. kashiwanohense* to consume LDFT and LNFP I has been recently suggested and might be a common characteristic of this species [40]. Not LN*n*T, nor 3′-sialyllactose (3′-SL), nor 6′-sialyllactose (6′-SL) were consumed by this species (Supplementary Figure S2) [70]. Assimilation ability for the other HMOs has not been described to our knowledge.

### 4.2. Two Different HMO Utilization Strategies and Prevalence of the Gene Set Required for HMO Assimilation

Infant gut-associated bifidobacteria have evolved the two different strategies for utilizing HMOs (intracellular and extracellular digestion strategies). *B. bifidum* and some *B. longum* strains use extracellular glycosidase(s) to liberate mono and/or disaccharides from HMOs outside the cells (Table 1, numbers 1–8). The liberated HMO degradants are subsequently imported by the cells and further degraded in the cytoplasm (e.g., Table 1, numbers 9, 10, and 27–30). In contrast, the other *Bifidobacterium* species/strains directly internalize intact HMOs by specific transporters (e.g., Table 1, numbers 11–13) and degrade them intracellularly (Table 1, numbers 14–26). The HMO utilization strategies employed by infant gut-associated bifidobacteria are schematically drawn in Figure 1 and Supplementary Figure S4.

**Figure 2:**
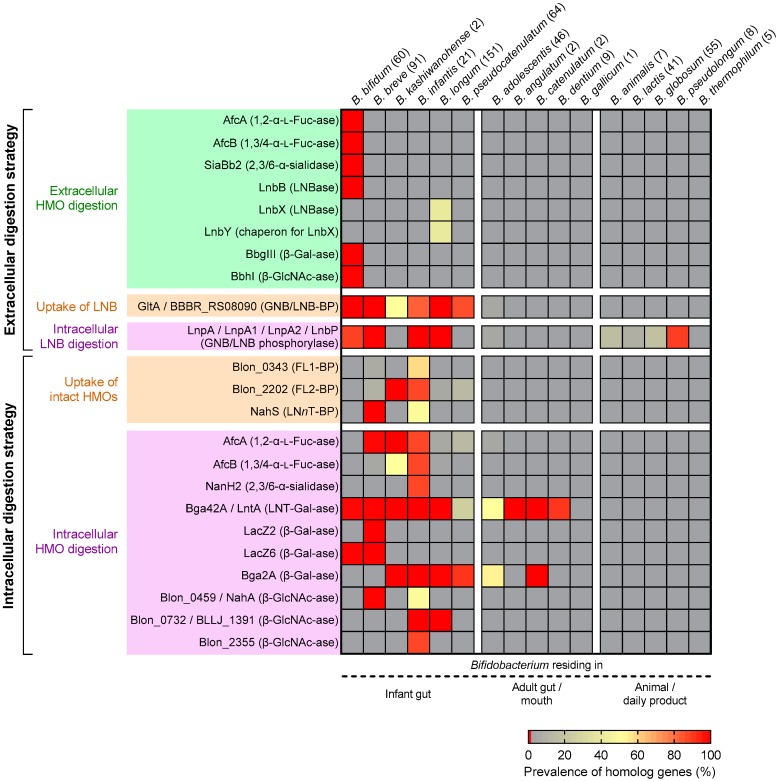
Prevalence of HMO-utilization genes in bifidobacterial genomes. Occurrence of the homolog genes (identity ≥70%, query coverage ≥60%, e value <1 × 10^−50^) in the sixteen *Bifidobacterium* (sub)species genomes was examined by tblastn analysis (BLAST+ v2.9.0). Query genes used in the analysis are shown in Table 1. The prevalence (%) of the genes in each (sub)species was determined by dividing the number of the retrieved genes with the above identity criteria by the number of the genomes examined (values in parentheses). The results are shown as a heatmap.

We carried out BLAST analysis to uncover the prevalence of the gene set required for HMO assimilation among *Bifidobacterium* species (Table 1). The species that are known to proliferate in infant guts, adult guts, or mouths, and non-human animal intestines or dairy products were chosen for the analysis (Figure 2). The results clearly showed that the HMO-related genes are almost exclusively found in the genomes of infant gut-associated *Bifidobacterium* species, and hardly detected in the genomes of other bifidobacterial species (Figure 2). The prevalence of the genes in the respective species varied significantly.

#### 4.2.1. Bifidobacterium Bifidum

*B. bifidum* (60 genomes examined) showed high conservation of all genes required for extracellular HMO degradation (note that LnbB and LnbXY catalyze the same reaction) (Figure 2). The results are consistent not only with the shared high HMO assimilation ability [32,58,66,67,69,70], but also with the shared cross-feeding ability of this species as described later (Section 7).

#### 4.2.2. Bifidobacterium Breve

As for *B. breve* (91 genomes examined), consistent with the finding that almost all strains of *B. breve* only utilize LNB, LNT, and LN*n*T [32,48,58,67,68,70], GNB/LNB-BP (GltA), LN*n*T-BP (NahS), and the intracellular enzymes required for degrading them (GNB/LNB phosphorylase, LNT β-1,3-galactosidase, two β-1,4-galactosidases (LacZ2/6), and one β-*N*-acetylglucosaminidase (NahA)) are highly conserved (Figure 2). *B. breve* commonly possesses an intracellular GH95 α-l-fucosidase, albeit that the prevalence of FL1-BP (4% occurrence) and FL2-BP (8% occurrence) is remarkably low. However, FL utilization ability and the presence of GH29 α-l-fucosidase has indeed been identified for several strains of *B. breve* [68].

#### 4.2.3. Bifidobacterium Longum Subsp. Infantis

The conservation of HMO-related genes in *B. infantis* genomes (21 examined) is very high, except for FL1-BP (57%), LN*n*T-BP (NahS) (48%), and β-*N*-acetylglucosaminidase (NahA) (48%) whose prevalence is moderate (Figure 2). However, the absence of FL1-BP and NahA was compensated for by the presence of FL2-BP (86% occurrence) and the other two GH20 β-*N*-acetylglucosaminidases (100% for Blon_0732 and 86% for Blon_2355).

#### 4.2.4. Bifidobacterium Longum Subsp. Longum

Extracellular GH136 lacto-*N*-biosidase (LnbX) is detected for 38% of *B. longum* genomes (151 examined) (Figure 2). LnbX-positive strains use GNB/LNB-BP (GltA) for LNB uptake and GNB/LNB phosphorylase (LnpA) for subsequent intracellular phosphorolysis, while LnbX-negative strains use LNT β-1,3-galactosidase for hydrolysis of LNT inside the cells. GltA, LnpA, GH42 LNT β-1,3-galactosidase, GH2 β-1,4-galactosidase (Bga2A homolog), and GH20 β-*N*-acetylglucosaminidase (Blon_0732 homolog) are highly conserved in *B. longum*, which is in agreement with the fact that this species commonly uses LNB and LNT [32,58,65,67]. Consistent with the observation that *B. longum* strains that are capable of utilizing fucosylated HMOs are rare, we found that the prevalence of FL2-BP (3% occurrence) and α-l-fucosidases (≤3%) was quite low.

#### 4.2.5. Bifidobacterium Kashiwanohense

The deposited two genomes of *B. kashiwanohense* was found to possess the homologs of FL2-BP, GH95 α-l-fucosidase (AfcA), LNT β-1,3-galactosidase, and one GH2 β-1,4-galactosidase (Bga2A) (Figure 2). The occurrence of GltA and GH29 α-l-fucosidase (AfcB) is strain-dependent, but GH20 β-*N*-acetylglucosaminidase gene homolog was not detected. LNT-assimilating ability has not been described for this species.

#### 4.2.6. Bifidobacterium Pseudocatenulatum

*B. pseudocatenulatum* (64 genomes examined) showed relatively high conservation of GNB/LNB transporter (GltA) and GH2 β-1,4-galactosidase (Bga2A) homolog genes (Figure 2). About half of the strains of those examined for this species were capable of assimilating LNB, as mentioned above [67], but lacked the GNB/LNB phosphorylase gene homolog. LNT β-1,3-galactosidase, which is moderately conserved in this species, may be involved in the disaccharide assimilation [51,67]. The FL2-BP (13%) and GH95 α-l-fucosidase (13%) homologs were sporadically detected, which is consistent with the report that several strains of this species utilize 2′-FL, 3-FL, and LDFT mixture as the carbon source [12].

#### 4.2.7. Other Bifidobacterium Species

The genomes of other *Bifidobacterium* species except for the above six (sub)species, which reside in adult guts or mouths, and non-human animal intestines or dairy products (176 genomes examined), were found to lack HMO degradation genes with a few exception (Figure 2). Indeed, some of these species showed poor growth on HMOs and LNB [12,43,67,72]. Instead, they appear to have gene sets for assimilating plant-derived glycans contained in adult or animal diet [73].

## 5. Abundance of HMO-Related Genes Is Associated with Bifidobacteria-Rich Microbiota Formation

Recently, Vatanen et al. [13,74] revealed, by fecal microbiome shotgun sequencing of the two different cohorts, that the HMO cluster homologs (Blon_2331–2361 of *B. infantis* ATCC 15697^T^) are enriched during breast-feeding, compared to after weaning. The results strongly suggest that the competitive advantage is conferred by the presence of the HMO cluster for bifidobacteria to dominate in the infant gut ecosystem. However, the increased fitness of bifidobacteria in the breast-fed infant gut microbial ecosystem should also be attributed to genes encoded in other loci. Our group recently showed that the FL1-BP and FL2-BP genes (Blon_0343 and Blon_2202 of *B. infantis* ATCC 15697^T^) are enriched in the fecal DNA of breast-fed infants and the relative abundance is positively correlated with the abundance of genus *Bifidobacterium* in the samples [40]. Interestingly, the abundance of the genes was negatively correlated with the fecal concentrations of fucosylated HMOs (2′-FL, 3-FL, LDFT, and/or LNFP I, the substrates of FL1-BP and FL2-BP). Thus, one interpretation of the results is that the bifidobacteria-rich microbiota is shaped by HMO consumption by *Bifidobacterium* in the breast-fed infant guts. The *lnbX* gene of *B. longum* was also found to be enriched in the exclusively breast-fed infant group, as compared with mixed (formula and breast)-fed infant group [60]. Given these findings, we analyzed the abundance of all the heretofore identified HMO utilization genes (enlisted in Table 1 and Figure 2) in a metagenomic database. 

**Figure 3:**
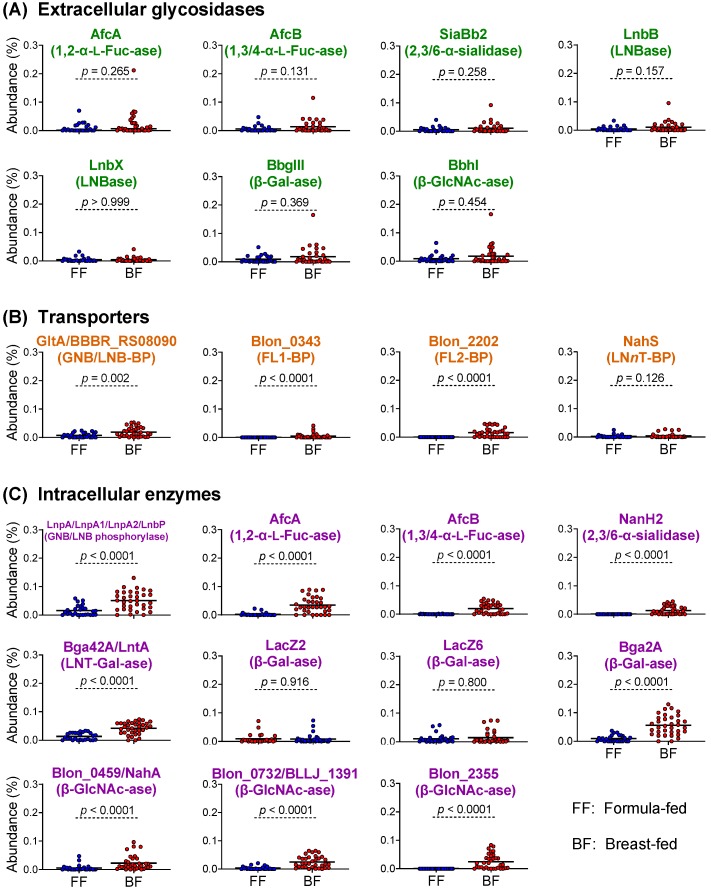
Metagenomic data mining analysis of HMO-related genes of bifidobacteria. (**A**–**C**) The abundances (%) of the genes for extracellular glycosidases (**A**), transporters (**B**), and intracellular enzymes (**C**) detected in the metagenomic data were compared between breast-fed (BF) and formula-fed (FF) infants (≤1 year old). Data from Yatsunenko et al. [39] (*n* = 27 for FF and *n* = 34 for BF; 117,492 ± 55,056 reads/sample) were used for the analysis. See Materials and Methods for calculation of the gene abundances. Mann–Whitney *U*-test was used for statistical significance evaluation.

The metagenomic dataset we analyzed (MG-RAST, accession number qiime:621) [39] includes 34 breast-fed and 27 formula-fed infants (≤1 year old) residing in the USA (*n* = 37), Malawi (*n* = 14), and Venezuela (*n* = 10). The data mining analysis revealed that the abundances of the extracellular glycosidase genes are indistinguishable in between the fecal DNAs obtained from formula-fed infants and breast-fed infants (Figure 3 and Supplementary Figure S5). By contrast, the abundances of the transporter and intracellular enzyme genes (for intracellular digestion strategy) were significantly higher in the breast-fed infant group than the formula-fed infant group, except for *nahS* (LN*n*T transporter), *lacZ2*, and *lacZ6* (GH2 β-1,4-galactosidases) (Figure 3; Mann–Whitney *U* test). The observed significant differences in the gene abundance between the two groups are mostly attributable to Malawian and Venezuelan breast-fed infants, which was revealed when the metagenomic reads were sorted out by the three countries (Supplementary Figure S5; Dunn’s test).

**Figure 4:**
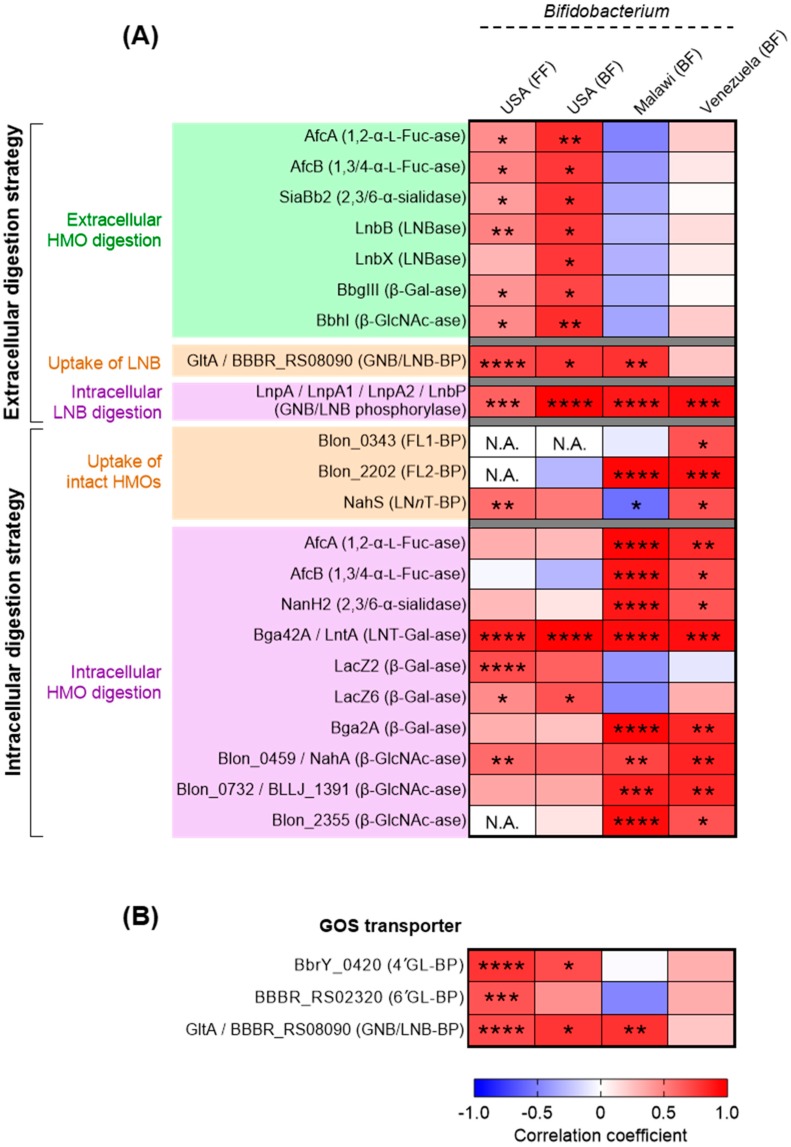
Heat map showing the results of Spearman’s rank correlation coefficient analysis between the relative abundance of genus *Bifidobacterium* and the relative abundance of HMO (**A**) or GOS utilization genes (**B**). The fecal metagenomic data of the infants living in USA, Malawi, and Venezuela were used for the analysis. The abundance of the genus *Bifidobacterium* was obtained from MG-RAST version 4.0.3 (see Material and Methods). See also, Supplementary Figure S5 for HMO degradation and transport genes (**A**) and Supplementary Figure S6 for GOS transporter genes (**B**). *, **, ***, and **** denote *p* < 0.05, *p* < 0.01, *p* < 0.001, and *p* < 0.0001 respectively. BF: breast-fed; FF: formula-fed; GL-BP: galactosyllactose-binding protein; N.A.: correlation analysis was not applicable due to the absence of the reads attributable to FL1/2-BP or GlcNAc-ase (Blon_2355) genes in the tested subjects.

We then analyzed the association between the relative abundance of respective HMO utilization genes and the relative abundance of genus *Bifidobacterium* in the infant stools (Figure 4A; Spearman’s rank correlation coefficient). The abundance of *Bifidobacterium* in the Malawian and Venezuelan breast-fed groups was positively correlated to the abundance of most of the genes responsible for the intracellular digestion strategy with statistical significances, while no strong positive or negative correlation was observed between the abundances of *Bifidobacterium* and the genes responsible for the extracellular digestion (Figure 4A). In contrast, in the breast-fed samples from the USA, the *Bifidobacterium* abundance was positively associated with the genes responsible for extracellular digestion (Figure 4A). In this group, relatively weak correlation was detected between the *Bifidobacterium* abundance and the genes responsible for intracellular digestion, although a strong positive correlation was observed for the LNT β-1,3-galactosidase gene that is highly conserved in infant gut-associated *Bifidobacterium* species (Figure 2). It should be noted the abundance of *Bifidobacterium* was essentially similar between the three country groups [40]. These results imply that bifidobacteria residing in the Malawian and Venezuelan infant guts mainly use the intracellular digestion strategy for HMO assimilation, while those colonizing USA infant intestines mainly adopt the extracellular digestion strategy (described later).

In the formula-fed infant group (the USA only), the abundance of *Bifidobacterium* appeared to show strong positive correlations with those of GOS transporter genes with statistical significance [47,75] (Figure 4B; Spearman’s rank correlation coefficient). GOSs are world-wide prebiotics supplemented in infant formula and are known to increase the population of bifidobacteria in the guts [76,77]. It is interesting to note that the abundances of the GOS transporter genes were similar between the formula-fed and breast-fed groups except for GNB/LNB-BP (GltA) gene (Supplementary Figure S6A,B); nonetheless, the slightly higher positive correlations were detected for 4′-galactosyllactose-SBP (4′GL-BP) and 6′-galactosyllactose-SBP (6′GL-BP) in infants who received formula milk than those receiving breast milk (Figure 4B and Supplementary Figure S6C) (note that sample numbers are different between the groups (formula-fed: *n* = 27, breast-fed: *n* = 10–14)). The GNB/LNB-BP (GltA) gene homolog is highly conserved in the genomes of infant gut-associated bifidobacteria (Figure 4B and Supplementary Figure S7).

## 6. Milk Oligosaccharide Composition May Dictate Transporter-Driven Bifidobacteria-Rich Microbiota Formation

HMO abundances and compositions vary among mothers, across geography, and between lactation stages within the same individual [23,24,25]. One of the most influential factors that give HMO structural variation between individuals is the maternal secretor status. In the mammary gland, fucosyltransferase-2 (FUT2) introduces α-1,2 linkage onto Gal residue (Fucα1-2Gal-*O*-R or H-antigen) at the non-reducing ends of milk glycans. Thus, HMOs synthesized by secretors (*FUT2^+/+^* or *FUT2^+/−^*) contain many H-antigen-carrying structures, such as 2′-FL, LDFT, LNFP I, and LNDFH I (≈50% of total HMOs by weight [25]), whereas those produced by non-secretors (*FUT2^−/−^*) do not. Considering that the conservation and prevalence of H-antigen-acting enzymes and transporters vary among infant gut-associated bifidobacterial species/strains (Table 1; Figure 1 and Figure 2), it is not surprising that the secretor status affects microbiota formation in infant guts. Indeed, Lewis et al. [34] reported that *Bifidobacterium* strains that are able to utilize 2′-FL are enriched in the stools of the infants receiving breast milk of secretors, compared to those of non-secretors. Several previous studies showed that the bifidobacterial population increases more rapidly and abundantly in infants fed by secretor mothers than those fed by non-secretor mothers [34,78,79]. Two FL transporters we mentioned above can import both 2′-FL and 3-FL [40]; therefore, bifidobacteria carrying the transporters can adapt to FL from both secretors and non-secretors. Nevertheless, the above results suggested that the secretors’ milk may be more advantageous for proliferating bifidobacteria. Detailed time-series analysis of the abundance of the gene set for HMO assimilation, as well as comparative studies of milk and fecal HMO concentration of mother-infant pairs during the lactation period, are necessary to understand the mechanistic differences in bifidobacteria-rich microbiota formation between infants receiving secretor’s milk and non-secretor’s milk. But, it is worth noting that FL2-BP (which binds to 2′FL and 3FL with equal affinity) is higher in abundance than FL1-BP (which preferentially binds to 2′FL over 3 FL), not only in breast-fed infant fecal metagenomes, but also in the genomes of infant gut-associated bifidobacteria (Figure 2 and Figure 3) [40]. Bifidobacteria may have evolved and shared FL transporter-2 to flexibly adapt to different host genetic statuses. The expansion of FL2-BP’s ability to additionally import LDFT and LNFP I may have occurred during the co-evolution of bifidobacteria with humans.

Given the importance of HMOs in the infant gut microbiota formation, industrial-scale production of HMOs has attracted significant attention in an effort to fortify infant formula [80]. Recently, 2′-FL and LN*n*T were approved for commercial supplementation. The safety, well-tolerance, and other benefits of the formula supplemented with 2′-FL (and LN*n*T) have been verified by several clinical trials [81,82]. These formulas may selectively promote the proliferation of bifidobacteria carrying either or both of FL and LN*n*T transporters. The results shown in Figure 4B might also provide evidence for the effectiveness of GOS supplementation to formula. The proposed transporter-driven bifidobacteria-rich microbiota formation is schematically presented in Supplementary Figure S8.

## 7. Cross-Feeding of HMO Degradants within *Bifidobacterium* Community

The transporter-dependent intracellular digestion strategy enables the bifidobacteria to efficiently capture preferred carbon sources in the competitive ecosystem over other gut microbes. However, in addition to this “selfish” strategy, bifidobacteria are considered to have another strategy to dominate the ecosystem; i.e., cross-feeding between the species/strains. Tannock et al. [83] examined the relationship between the presence of *B. bifidum* and the dominance of genus *Bifidobacterium* in the infant guts. Interestingly, they found that, when *B. bifidum* occupies >10% of genus *Bifidobacterium*, the corresponding microbiota possesses a higher ratio of *Bifidobacterium* abundance than those containing *B. bifidum* of less than 10% population among the genus. The predominance of genus *Bifidobacterium* in the presence of adequate amount of *B. bifidum* was observed only for breast-fed infant stools but not for cow-based formula milk-fed infant feces. Before their report, we had noticed that *B. bifidum* leaves HMO degradants unconsumed in the spent media, even after prolonged incubation on HMOs [58]. These results suggested that HMO degradants produced by *B. bifidum* extracellularly can be shared within bifidobacterial community.

SL-mediated cross-feeding between *B. bifidum* and *B. breve* was shown by Egan et al. [84] and Nishiyama et al. [85] in co-culture experiments. In these cases, Neu5Ac liberated from SL by extracellular sialidase SiaBb2 of *B. bifidum* was utilized by *B. breve* carrying the *nan* cluster that is responsible for converting Neu5Ac into pyruvic acid and GlcNAc 6-phosphate with one ATP consumption.

When stool suspensions obtained from infants, children, and adults were cultured in the media supplemented HMOs in the presence and absence of *B. bifidum*, a remarkable difference was observed: i.e., the population of *Bifidobacterium* species other than *B. bifidum* significantly increased when *B. bifidum* was added prior to cultivation, as compared with non-added control [66]. Importantly, the relative abundance (%) of other *Bifidobacterium* species in the total bacterial community was increased as well in the fecal cultures supplemented with *B. bifidum*. On the other hand, in the stool samples grown on Glc, neither the relative abundance nor population of other *Bifidobacterium* species was significantly changed by the addition of *B. bifidum*. In addition, the growth stimulatory effect of *B. bifidum* on other *Bifidobacterium* species observed for HMO-supplemented fecal cultures disappeared when fuconojirimycin, a potent fucosidase inhibitor (both for GH29 and GH95), was added to the media [57,66]. Bifidobacteria-rich gut microbiota observed for USA infants that are associated with the abundance of extracellular digestion enzymes (Figure 4), thus, appears to be established by cross-feeding by *B. bifidum*. These “altruistic” behaviors might be a common characteristic of *B. bifidum.*

## 8. Conclusions

In the past 15 years, a series of the genes that are involved in HMO degradation and transport have been identified and characterized from bifidobacteria (Table 1), which led to the findings that these genes are almost exclusively present in the genomes of infant gut-associated bifidobacteria (Figure 2). However, there have been no reports that systematically analyzed the conservation of genes among *Bifidobacterium* spp. In this review, we analyzed bifidobacterial genomes to understand the prevalence of the HMO-related gene set at the species and strain levels. We also conducted in silico data mining analysis using an infant fecal metagenomic dataset to examine how the abundances of the HMO-related genes are linked with bifidobacteria-rich microbiota formation. The results obtained were then discussed based on the HMO assimilation mechanism that each *Bifidobacterium* has evolved (Figure 1 and Supplementary Figure S4). Although our knowledge of HMO transporters is still incomplete, the presented data could dictate the importance that HMO assimilation pathways play in the formation of the infant gut microbiota.

Recent reports strongly suggest that in vivo consumption of HMOs is associated with bifidobacteria-rich microbiota formation in infant guts [12,34,35,36,37]. Frese et al. [86] reported that continuous administration of *B. infantis* EVC001 to the breast-fed infants from day 7 to day 28 led to the decrease of fecal HMO concentrations in infant stools, which coincided with increased fecal abundance (>10^10^ cells/g feces) of the strain. A similar observation was also reported by the same research group using *B. breve* M-16V [87]. Interestingly, in the former case, the fecal concentrations of most HMOs, including fucosylated ones, were decreased, whereas in the latter case, fecal consumption of HMOs was essentially limited to undecorated (non-fucosylated) HMOs. The two studies were conducted in different cohorts, and thus it is not appropriately comparable; however, it seems that the observed difference in the fecal HMO consumption behavior between the two cohorts may reflect the difference in the possession of HMO-related gene set of between *B. infantis* and *B. breve* (Figure 1 and Figure 2).

Apparently, the formation of the infant gut microbiome is affected by the level of HMO consumption by bifidobacteria in infant guts. However, we must understand the extent to which endogenous (or exogenously supplemented) bifidobacterial species/strains that possess variable gene sets are responsible for HMO assimilation in order to gain mechanistic insight into bifidobacterial-rich microbiota formation in the guts. Such understanding helps us not only to understand the results obtained in clinical studies but also to design future clinical interventions that elucidate the effects of supplementation of HMOs and probiotic *Bifidobacterium* strains in formula.

Besides infant gut-associated bifidobacteria, it has been reported that a few other gut microbes belonging to different taxa have HMO consumption abilities. Several *Bacteroides* species were shown to assimilate 2′-FL, 3-FL, LDFT, 3′-SL, and 6′-SL [88]. *Lactobacillus casei* utilizes HMO degradants LNTri II and LNB [89,90]. Interestingly, *Lactobacillus* has evolved a specific phosphotransferase system to import LNB [90]. HMO utilization capability of these gut microbes is generally not very high, but it is worth exploring how minority species during the breast-feeding stage persist until the introduction of solid food (weaning), which potentially influences maturation of the microbiota in adulthood.

## Figures and Tables

**Table 1:** Human milk oligosaccharide (HMO)-related bifidobacterial enzymes and transporters described in this study.

No ^1^	Protein Name	Locus Tag ^2^	Abbreviation ^2^	Origin	GenBank Accession No.	Reference
*Extracellular glycosidases*						
1	1,2-α-l-Fucosidase	N.A.	AfcA	*B. bifidum* JCM 1254	AY303700.1	[27]
2	1,3/4-α-l-Fucosidase	N.A.	AfcB	*B. bifidum* JCM 1254	AB474964.1	[41]
3	2,3/6-α-Sialidase	N.A.	SiaBb2	*B. bifidum* JCM 1254	AB278567.1	[42]
4	Lacto-*N*-biosidase	N.A.	LnbB	*B. bifidum* JCM 1254	EU281545.1	[43]
5	Lacto-*N*-biosidase	BLLJ_1505	LnbX	*B. longum* JCM 1217^T^	AP010888.1	[44]
6	Chaperon for LnbX	BLLJ_1506	LnbY	*B. longum* JCM 1217^T^	AP010888.1	[44]
7	β-1,4-Galactosidase	N.A.	BbgIII	*B. bifidum* JCM 1254	AB504520.1	[45]
8	β-*N*-Acetylglucosaminidase	N.A.	BbhI	*B. bifidum* JCM 1254	AB504521.1	[45]
*Transporters*						
9	GNB/LNB transporter SBP	BLLJ_1626	GltA	*B. longum* JCM 1217^T^	AP010888.1	[46]
10	GNB/LNB transporter SBP	BBBR_RS08090	N.A.	*B. breve* YIT 4014^T^	LC333766.1	[47]
11	FL transporter SBP	Blon_0343	FL1-BP	*B. infantis* ATCC 15697^T^	CP001095.1	[40]
12	FL transporter SBP	Blon_2202	FL2-BP	*B. infantis* ATCC 15697^T^	CP001095.1	[40]
13	LN*n*T transporter SBP	Bbr_1554	NahS	*B. breve* UCC2003	CP000303.1	[48]
*Intracellular enzymes*						
14	1,2-α-l-Fucosidase	Blon_2335	AfcA	*B. infantis* ATCC 15697^T^	CP001095.1	[49]
15	1,3/4-α-l-Fucosidase	Blon_2336	AfcB	*B. infantis* ATCC 15697^T^	CP001095.1	[49]
16	2,3/6-α-Sialidase	Blon_2348	NanH2	*B. infantis* ATCC 15697^T^	CP001095.1	[50]
17	LNT β-1,3-Galactosidase	Bbr_0529	LntA	*B. breve* UCC2003	CP000303.1	[48]
18	LNT β-1,3-Galactosidase	Blon_2016	Bga42A	*B. infantis* ATCC 15697^T^	CP001095.1	[51]
19	β-1,4-Galactosidase	Blon_2334	Bga2A	*B. infantis* ATCC 15697^T^	CP001095.1	[51]
20	β-1,4-Galactosidase	Bbr_0010	LacZ2	*B. breve* UCC2003	CP000303.1	[48]
21	β-1,4-Galactosidase	Bbr_1552	LacZ6	*B. breve* UCC2003	CP000303.1	[48]
22	β-*N*-Acetylglucosaminidase	Bbr_1556	NahA	*B. breve* UCC2003	CP000303.1	[48]
23	β-*N*-Acetylglucosaminidase	BLLJ_1391	N.A.	*B. longum* JCM 1217^T^	BAJ67058.1	[52]
24	β-*N*-Acetylglucosaminidase	Blon_0459	N.A.	*B. infantis* ATCC 15697^T^	CP001095.1	[53]
25	β-*N*-Acetylglucosaminidase	Blon_0732	N.A.	*B. infantis* ATCC 15697^T^	CP001095.1	[53]
26	β-*N*-Acetylglucosaminidase	Blon_2355	N.A.	*B. infantis* ATCC 15697^T^	CP001095.1	[53]
27	GNB/LNB phosphorylase	N.A.	LnpA1	*B. bifidum* JCM 1254	AB181927.1	[54]
28	GNB/LNB phosphorylase	N.A.	LnpA2	*B. bifidum* JCM 1254	AB262011.1	[54]
29	GNB/LNB phosphorylase	Bbr_1587	LnbP	*B. breve* UCC2003	CP000303.1	[48]
30	GNB/LNB phosphorylase	BLLJ_1623	LnpA	*B. longum* JCM 1217^T^	AP010888.1	[28]

^1^ The numbers for query genes used in the analysis are underlined. ^2^ N.A.: not applicable.

## Supplementary Materials

The following are available online at https://www.mdpi.com/2072-6643/12/1/71/s1. Figure S1: Graphical abstract of the in silico analysis procedures adopted in this study. Figure S2: Representative twelve HMO molecules described in this study, and the HMO degradants produced in the assimilation pathways. Figure S3: Unique specificity observed for α-l-fucosidases can be explained by the structural similarities between the different substrates. Figure S4: HMO utilization pathways of *B. pseudocatenulatum* and *B. kashiwanohense*. Figure S5: Metagenomic data mining analysis of HMO-related genes of bifidobacteria. Figure S6: Metagenomic data mining analysis of GOS transporter homolog genes in bifidobacteria. Figure S7: Prevalence of GOS transporter genes in the genomes of bifidobacteria. Figure S8: Bifidobacteria-rich microbiota formation processes in infant guts can differ between feeding strategies and be partially dependent on specific transporters. Table S1: The accession numbers of *Bifidobacterium* strains used for the in silico analysis.

## References

[1] Mattarelli P., Biavati B., Mattarelli P., Biavati B., Holzapfel W.H., Wood B.J.B. (2018). Species in the genus Bifidobacterium. The Bifidobacteria and Related Organisms: Biology, Taxonomy, Applications.

[2] Pechar R., Killer J., Salmonová H., Geigerová M., Švejstil R., Švec P., Sedláček I., Rada V., Benada O. (2017). *Bifidobacterium apri* sp. nov., a thermophilic actinobacterium isolated from the digestive tract of wild pigs (*Sus scrofa*). Int. J. Syst. Evol. Microbiol..

[3] Modesto M., Watanabe K., Arita M., Satti M., Oki K., Sciavilla P., Patavino C., Cammà C., Michelini S., Sgorbati B. (2019). *Bifidobacterium jacchi* sp. nov., isolated from the faeces of a baby common marmoset (*Callithrix jacchus*). Int. J. Syst. Evol. Microbiol..

[4] Modesto M., Satti M., Watanabe K., Puglisi E., Morelli L., Huang C.H., Liou J.S., Miyashita M., Tamura T., Saito S. (2019). Characterization of *Bifidobacterium* species in feaces of the Egyptian fruit bat: Description of *B. vespertilionis* sp. nov. and *B. rousetti* sp. nov. Syst. Appl. Microbiol..

[5] Modesto M., Puglisi E., Bonetti A., Michelini S., Spiezio C., Sandri C., Sgorbati B., Morelli L., Mattarelli P. (2018). *Bifidobacterium primatium* sp. nov., *Bifidobacterium scaligerum* sp. nov., *Bifidobacterium felsineum* sp. nov. and *Bifidobacterium simiarum* sp. nov.: Four novel taxa isolated from the faeces of the cotton top tamarin (*Saguinus oedipus*) and the emperor tamarin (*Saguinus imperator*). Syst. Appl. Microbiol..

[6] Modesto M., Michelini S., Sansosti M.C., De Filippo C., Cavalieri D., Qvirist L., Andlid T., Spiezio C., Sandri C., Pascarelli S. (2018). *Bifidobacterium callitrichidarum* sp. nov. from the faeces of the emperor tamarin (*Saguinus imperator*). Int. J. Syst. Evol. Microbiol..

[7] Modesto M., Michelini S., Oki K., Biavati B., Watanabe K., Mattarelli P. (2018). *Bifidobacterium catulorum* sp. nov., a novel taxon from the faeces of the baby common marmoset (*Callithrix jacchus*). Int. J. Syst. Evol. Microbiol..

[8] Lugli G.A., Mangifesta M., Duranti S., Anzalone R., Milani C., Mancabelli L., Alessandri G., Turroni F., Ossiprandi M.C., van Sinderen D. (2018). Phylogenetic classification of six novel species belonging to the genus *Bifidobacterium* comprising *Bifidobacterium anseris* sp. nov., *Bifidobacterium criceti* sp. nov., *Bifidobacterium imperatoris* sp. nov., *Bifidobacterium italicum* sp. nov., *Bifidobacterium margollesii* sp. nov. and *Bifidobacterium parmae* sp. nov. Syst. Appl. Microbiol..

[9] Duranti S., Mangifesta M., Lugli G.A., Turroni F., Anzalone R., Milani C., Mancabelli L., Ossiprandi M.C., Ventura M. (2017). *Bifidobacterium vansinderenii* sp. nov., isolated from faeces of emperor tamarin (*Saguinus imperator*). Int. J. Syst. Evol. Microbiol..

[10] Duranti S., Lugli G.A., Napoli S., Anzalone R., Milani C., Mancabelli L., Alessandri G., Turroni F., Ossiprandi M.C., van Sinderen D. (2019). Characterization of the phylogenetic diversity of five novel species belonging to the genus *Bifidobacterium*: *Bifidobacterium castoris* sp. nov., *Bifidobacterium callimiconis* sp. nov., *Bifidobacterium goeldii* sp. nov., *Bifidobacterium samirii* sp. nov. and *Bifidobacterium dolichotidis* sp. nov. Int. J. Syst. Evol. Microbiol..

[11] Alberoni D., Gaggìa F., Baffoni L., Modesto M.M., Biavati B., Di Gioia D. (2019). *Bifidobacterium xylocopae* sp. nov. and *Bifidobacterium aemilianum* sp. nov., from the carpenter bee (*Xylocopa violacea*) digestive tract. Syst. Appl. Microbiol..

[12] Matsuki T., Yahagi K., Mori H., Matsumoto H., Hara T., Tajima S., Ogawa E., Kodama H., Yamamoto K., Yamada T. (2016). A key genetic factor for fucosyllactose utilization affects infant gut microbiota development. Nat. Commun..

[13] Vatanen T., Franzosa E.A., Schwager R., Tripathi S., Arthur T.D., Vehik K., Lernmark Å., Hagopian W.A., Rewers M.J., She J.X. (2018). The human gut microbiome in early-onset type 1 diabetes from the TEDDY study. Nature.

[14] Stewart C.J., Ajami N.J., O’Brien J.L., Hutchinson D.S., Smith D.P., Wong M.C., Ross M.C., Lloyd R.E., Doddapaneni H., Metcalf G.A. (2018). Temporal development of the gut microbiome in early childhood from the TEDDY study. Nature.

[15] Sugahara H., Odamaki T., Hashikura N., Abe F., Xiao J.Z. (2015). Differences in folate production by bifidobacteria of different origins. Biosci. Microbiota Food Health.

[16] Huda M.N., Lewis Z., Kalanetra K.M., Rashid M., Ahmad S.M., Raqib R., Qadri F., Underwood M.A., Mills D.A., Stephensen C.B. (2014). Stool microbiota and vaccine responses of infants. Pediatrics.

[17] Kalliomäki M., Kirjavainen P., Eerola E., Kero P., Salminen S., Isolauri E. (2001). Distinct patterns of neonatal gut microflora in infants in whom atopy was and was not developing. J. Allergy Clin. Immunol..

[18] O’Connell-Motherway M., Houston A., O’Callaghan G., Reunanen J., O’Brien F., O’Driscoll T., Casey P.G., de Vos W.M., van Sinderen D., Shanahan F. (2019). A bifidobacterial pilus-associated protein promotes colonic epithelial proliferation. Mol. Microbiol..

[19] Odamaki T., Kato K., Sugahara H., Hashikura N., Takahashi S., Xiao J.Z., Abe F., Osawa R. (2016). Age-related changes in gut microbiota composition from newborn to centenarian: A cross-sectional study. BMC Microbiol..

[20] Kato K., Odamaki T., Mitsuyama E., Sugahara H., Xiao J.Z., Osawa R. (2017). Age-related changes in the composition of gut *Bifidobacterium* species. Curr. Microbiol..

[21] György P., Kuhn R., Rose C.S., Zilliken F. (1954). Bifidus factor. II. Its occurrence in milk from different species and in other natural products. Arch. Biochem. Biophys..

[22] Gauhe A., György P., Hoover J.R.E., Kuhn R., Rose C.S., Ruelius H.W., Zilliken F. (1954). Bifidus factor. IV. Preparations obtained from human milk. Arch. Biochem. Biophys..

[23] Urashima T., Asakuma S., Leo F., Fukuda K., Messer M., Oftedal O.T. (2012). The predominance of type I oligosaccharides is a feature specific to human breast milk. Adv. Nutr..

[24] Kunz C., Rudloff S., Baier W., Klein N., Strobel S. (2000). Oligosaccharides in human milk: Structural, functional, and metabolic aspects. Annu. Rev. Nutr..

[25] McGuire M.K., Meehan C.L., McGuire M.A., Williams J.E., Foster J., Sellen D.W., Kamau-Mbuthia E.W., Kamundia E.W., Mbugua S., Moore S.E. (2017). What’s normal? Oligosaccharide concentrations and profiles in milk produced by healthy women vary geographically. Am. J. Clin. Nutr..

[26] Glick M.C., Sall T., Zilliken F., Mudd S. (1960). Morphological changes of *Lactobacillus bifidus* var. *pennsylvanicus* produced by a cell-wall precursor. Biochim. Biophys. Acta.

[27] Katayama T., Sakuma A., Kimura T., Makimura Y., Hiratake J., Sakata K., Yamanoi T., Kumagai H., Yamamoto K. (2004). Molecular cloning and characterization of *Bifidobacterium bifidum* 1,2-α-l-fucosidase (AfcA), a novel inverting glycosidase (glycoside hydrolase family 95). J. Bacteriol..

[28] Kitaoka M., Tian J., Nishimoto M. (2005). Novel putative galactose operon involving lacto-*N*-biose phosphorylase in *Bifidobacterium longum*. Appl. Environ. Microbiol..

[29] Ward R.E., Niñonuevo M., Mills D.A., Lebrilla C.B., German J.B. (2006). In vitro fermentation of breast milk oligosaccharides by *Bifidobacterium infantis* and *Lactobacillus gasseri*. Appl. Environ. Microbiol..

[30] Sela D.A., Chapman J., Adeuya A., Kim J.H., Chen F., Whitehead T.R., Lapidus A., Rokhsar D.S., Lebrilla C.B., German J.B. (2008). The genome sequence of *Bifidobacterium longum* subsp. *infantis* reveals adaptations for milk utilization within the infant microbiome. Proc. Natl. Acad. Sci. USA.

[31] Katayama T. (2016). Host-derived glycans serve as selected nutrients for the gut microbe: Human milk oligosaccharides and bifidobacteria. Biosci. Biotechnol. Biochem..

[32] Thomson P., Medina D.A., Garrido D. (2018). Human milk oligosaccharides and infant gut bifidobacteria: Molecular strategies for their utilization. Food Microbiol..

[33] Zúñiga M., Monedero V., Yebra M.J. (2018). Utilization of host-derived glycans by intestinal *Lactobacillus* and *Bifidobacterium* species. Front. Microbiol..

[34] Lewis Z.T., Totten S.M., Smilowitz J.T., Popovic M., Parker E., Lemay D.G., Van Tassell M.L., Miller M.J., Jin Y.S., German J.B. (2015). Maternal fucosyltransferase 2 status affects the gut bifidobacterial communities of breastfed infants. Microbiome.

[35] Borewicz K., Gu F., Saccenti E., Arts I.C.W., Penders J., Thijs C., van Leeuwen S.S., Lindner C., Nauta A., van Leusen E. (2019). Correlating infant faecal microbiota composition and human milk oligosaccharide consumption by microbiota of one-month old breastfed infants. Mol. Nutr. Food Res..

[36] Davis J.C.C., Totten S.M., Huang J.O., Nagshbandi S., Kirmiz N., Garrido D.A., Lewis Z.T., Wu L.D., Smilowitz J.T., German J.B. (2016). Identification of oligosaccharides in feces of breast-fed infants and their correlation with the gut microbial community. Mol. Cell. Proteom..

[37] De Leoz M.L.A., Kalanetra K.M., Bokulich N.A., Strum J.S., Underwood M.A., German J.B., Mills D.A., Lebrilla C.B. (2015). Human milk glycomics and gut microbial genomics in infant feces show a correlation between human milk oligosaccharides and gut microbiota: A proof-of-concept study. J. Proteome Res..

[38] Walker W.A., Iyengar R.S. (2015). Breast milk, microbiota, and intestinal immune homeostasis. Pediatr. Res..

[39] Yatsunenko T., Rey F.E., Manary M.J., Trehan I., Dominguez-Bello M.G., Contreras M., Magris M., Hidalgo G., Baldassano R.N., Anokhin A.P. (2012). Human gut microbiome viewed across age and geography. Nature.

[40] Sakanaka M., Hansen M.E., Gotoh A., Katoh T., Yoshida K., Odamaki T., Yachi H., Sugiyama Y., Kurihara S., Hirose J. (2019). Evolutionary adaptation in fucosyllactose uptake systems supports bifidobacteria-infant symbiosis. Sci. Adv..

[41] Ashida H., Miyake A., Kiyohara M., Wada J., Yoshida E., Kumagai H., Katayama T., Yamamoto K. (2009). Two distinct α-l-fucosidases from *Bifidobacterium bifidum* are essential for the utilization of fucosylated milk oligosaccharides and glycoconjugates. Glycobiology.

[42] Kiyohara M., Tanigawa K., Chaiwangsri T., Katayama T., Ashida H., Yamamoto K. (2011). An exo-α-sialidase from bifidobacteria involved in the degradation of sialyloligosaccharides in human milk and intestinal glycoconjugates. Glycobiology.

[43] Wada J., Ando T., Kiyohara M., Ashida H., Kitaoka M., Yamaguchi M., Kumagai H., Katayama T., Yamamoto K. (2008). *Bifidobacterium bifidum* lacto-*N*-biosidase, a critical enzyme for the degradation of human milk oligosaccharides with a type 1 structure. Appl. Environ. Microbiol..

[44] Sakurama H., Kiyohara M., Wada J., Honda Y., Yamaguchi M., Fukiya S., Yokota A., Ashida H., Kumagai H., Kitaoka M. (2013). Lacto-*N*-biosidase encoded by a novel gene of *Bifidobacterium longum* subspecies *longum* shows unique substrate specificity and requires a designated chaperone for its active expression. J. Biol. Chem..

[45] Miwa M., Horimoto T., Kiyohara M., Katayama T., Kitaoka M., Ashida H., Yamamoto K. (2010). Cooperation of β-galactosidase and β-*N*-acetylhexosaminidase from bifidobacteria in assimilation of human milk oligosaccharides with type 2 structure. Glycobiology.

[46] Suzuki R., Wada J., Katayama T., Fushinobu S., Wakagi T., Shoun H., Sugimoto H., Tanaka A., Kumagai H., Ashida H. (2008). Structural and thermodynamic analyses of solute-binding protein from *Bifidobacterium longum* specific for core 1 disaccharide and lacto-*N*-biose I. J. Biol. Chem..

[47] Sotoya H., Shigehisa A., Hara T., Matsumoto H., Hatano H., Matsuki T. (2017). Identification of genes involved in galactooligosaccharide utilization in *Bifidobacterium breve* strain YIT 4014^T^. Microbiology.

[48] James K., O’Connell-Motherway M., Bottacini F., van Sinderen D. (2016). *Bifidobacterium breve* UCC2003 metabolises the human milk oligosaccharides lacto-*N*-tetraose and lacto-*N*-*neo*-tetraose through overlapping, yet distinct pathways. Sci. Rep..

[49] Sela D.A., Garrido D., Lerno L., Wu S., Tan K., Eom H.J., Joachimiak A., Lebrilla C.B., Mills D.A. (2012). *Bifidobacterium longum* subsp. *infantis* ATCC 15697 α-fucosidases are active on fucosylated human milk oligosaccharides. Appl. Environ. Microbiol..

[50] Sela D.A., Li Y., Lerno L., Wu S., Marcobal A.M., German J.B., Chen X., Lebrilla C.B., Mills D.A. (2011). An infant-associated bacterial commensal utilizes breast milk sialyloligosaccharides. J. Biol. Chem..

[51] Yoshida E., Sakurama H., Kiyohara M., Nakajima M., Kitaoka M., Ashida H., Hirose J., Katayama T., Yamamoto K., Kumagai H. (2012). *Bifidobacterium longum* subsp. *infantis* uses two different β-galactosidases for selectively degrading type-1 and type-2 human milk oligosaccharides. Glycobiology.

[52] Honda Y., Nishimoto M., Katayama T., Kitaoka M. (2013). Characterization of the cytosolic β-*N*-acetylglucosaminidase from *Bifidobacterium longum* subsp. longum. J. Appl. Glycosi..

[53] Garrido D., Ruiz-Moyano S., Mills D.A. (2012). Release and utilization of *N*-acetyl-d-glucosamine from human milk oligosaccharides by *Bifidobacterium longum* subsp. infantis. Anaerobe.

[54] Nishimoto M., Kitaoka M. (2007). Identification of the putative proton donor residue of lacto-*N*-biose phosphorylase (EC 2.4.1.211). Biosci. Biotechnol. Biochem..

[55] Henrissat B., Davies G. (1997). Structural and sequence-based classification of glycoside hydrolases. Curr. Opin. Struct. Biol..

[56] Sakurama H., Fushinobu S., Hidaka M., Yoshida E., Honda Y., Ashida H., Kitaoka M., Kumagai H., Yamamoto K., Katayama T. (2012). 1,3-1,4-α-l-fucosynthase that specifically introduces Lewis a/x antigens into type-1/2 chains. J. Biol. Chem..

[57] Nagae M., Tsuchiya A., Katayama T., Yamamoto K., Wakatsuki S., Kato R. (2007). Structural basis of the catalytic reaction mechanism of novel 1,2-α-l-fucosidase from *Bifidobacterium bifidum*. J. Biol. Chem..

[58] Asakuma S., Hatakeyama E., Urashima T., Yoshida E., Katayama T., Yamamoto K., Kumagai H., Ashida H., Hirose J., Kitaoka M. (2011). Physiology of consumption of human milk oligosaccharides by infant gut-associated bifidobacteria. J. Biol. Chem..

[59] Nishiyama K., Yamamoto Y., Sugiyama M., Takaki T., Urashima T., Fukiya S., Yokota A., Okada N., Mukai T. (2017). *Bifidobacterium bifidum* extracellular sialidase enhances adhesion to the mucosal surface and supports carbohydrate assimilation. mBio.

[60] Yamada C., Gotoh A., Sakanaka M., Hattie M., Stubbs K.A., Katayama-Ikegami A., Hirose J., Kurihara S., Arakawa T., Kitaoka M. (2017). Molecular insight into evolution of symbiosis between breast-fed infants and a member of the human gut microbiome *Bifidobacterium longum*. Cell Chem. Biol..

[61] Gotoh A., Katoh T., Sugiyama Y., Kurihara S., Honda Y., Sakurama H., Kambe T., Ashida H., Kitaoka M., Yamamoto K. (2015). Novel substrate specificities of two lacto-*N*-biosidases towards β-linked galacto-*N*-biose-containing oligosaccharides of globo H, Gb5, and GA1. Carbohydr. Res..

[62] Garrido D., Kim J.H., German J.B., Raybould H.E., Mills D.A. (2011). Oligosaccharide binding proteins from *Bifidobacterium longum* subsp. *infantis* reveal a preference for host glycans. PLoS ONE.

[63] Fujita K., Oura F., Nagamine N., Katayama T., Hiratake J., Sakata K., Kumagai H., Yamamoto K. (2005). Identification and molecular cloning of a novel glycoside hydrolase family of core 1 type *O*-glycan-specific endo-α-*N*-acetylgalactosaminidase from *Bifidobacterium longum*. J. Biol. Chem..

[64] Wada J. (2009). Studies on Human Milk Oligosaccharide Metabolism in Bifidobacteria. Ph.D. Thesis.

[65] Garrido D., Ruiz-Moyano S., Kirmiz N., Davis J.C., Totten S.M., Lemay D.G., Ugalde J.A., German J.B., Lebrilla C.B., Mills D.A. (2016). A novel gene cluster allows preferential utilization of fucosylated milk oligosaccharides in *Bifidobacterium longum* subsp. longum SC596. Sci. Rep..

[66] Gotoh A., Katoh T., Sakanaka M., Ling Y., Yamada C., Asakuma S., Urashima T., Tomabechi Y., Katayama-Ikegami A., Kurihara S. (2018). Sharing of human milk oligosaccharides degradants within bifidobacterial communities in faecal cultures supplemented with *Bifidobacterium bifidum*. Sci. Rep..

[67] Xiao J.Z., Takahashi S., Nishimoto M., Odamaki T., Yaeshima T., Iwatsuki K., Kitaoka M. (2010). Distribution of *in vitro* fermentation ability of lacto-*N*-biose I, a major building block of human milk oligosaccharides, in bifidobacterial strains. Appl. Environ. Microbiol..

[68] Ruiz-Moyano S., Totten S.M., Garrido D.A., Smilowitz J.T., German J.B., Lebrilla C.B., Mills D.A. (2013). Variation in consumption of human milk oligosaccharides by infant gut-associated strains of *Bifidobacterium breve*. Appl. Environ. Microbiol..

[69] Garrido D., Ruiz-Moyano S., Lemay D.G., Sela D.A., German J.B., Mills D.A. (2015). Comparative transcriptomics reveals key differences in the response to milk oligosaccharides of infant gut-associated bifidobacteria. Sci. Rep..

[70] Bunesova V., Lacroix C., Schwab C. (2016). Fucosyllactose and l-fucose utilization of infant *Bifidobacterium longum* and *Bifidobacterium kashiwanohense*. BMC Microbiol..

[71] LoCascio R.G., Desai P., Sela D.A., Weimer B., Mills D.A. (2010). Broad conservation of milk utilization genes in *Bifidobacterium longum* subsp. *infantis* as revealed by comparative genomic hybridization. Appl. Environ. Microbiol..

[72] LoCascio R.G., Niñonuevo M.R., Kronewitter S.R., Freeman S.L., German J.B., Lebrilla C.B., Mills D.A. (2009). A versatile and scalable strategy for glycoprofiling bifidobacterial consumption of human milk oligosaccharides. Microb. Biotechnol..

[73] Milani C., Turroni F., Duranti S., Lugli G.A., Mancabelli L., Ferrario C., van Sinderen D., Ventura M. (2016). Genomics of the genus *Bifidobacterium* reveals species-specific adaptation to the glycan-rich gut environment. Appl. Environ. Microbiol..

[74] Vatanen T., Plichta D.R., Somani J., Münch P.C., Arthur T.D., Hall A.B., Rudolf S., Oakeley E.J., Ke X., Young R.A. (2019). Genomic variation and strain-specific functional adaptation in the human gut microbiome during early life. Nat. Microbiol..

[75] Shigehisa A., Sotoya H., Sato T., Hara T., Matsumoto H., Matsuki T. (2015). Characterization of a bifidobacterial system that utilizes galacto-oligosaccharides. Microbiology.

[76] Sierra C., Bernal M.J., Blasco J., Martínez R., Dalmau J., Ortuño I., Espín B., Vasallo M.I., Gil D., Vidal M.L. (2015). Prebiotic effect during the first year of life in healthy infants fed formula containing GOS as the only prebiotic: A multicentre, randomised, double-blind and placebo-controlled trial. Eur. J. Nutr..

[77] Matsuki T., Tajima S., Hara T., Yahagi K., Ogawa E., Kodama H. (2016). Infant formula with galacto-oligosaccharides (OM55N) stimulates the growth of indigenous bifidobacteria in healthy term infants. Benef. Microbes.

[78] Lewis Z.T., Sidamonidze K., Tsaturyan V., Tsereteli D., Khachidze N., Pepoyan A., Zhgenti E., Tevzadze L., Manvelyan A., Balayan M. (2017). The fecal microbial community of breast-fed infants from Armenia and Georgia. Sci. Rep..

[79] Bai Y., Tao J., Zhou J., Fan Q., Liu M., Hu Y., Xu Y., Zhang L., Yuan J., Li W. (2018). Fucosylated human milk oligosaccharides and *N*-glycans in the milk of Chinese mothers regulate the gut microbiome of their breast-fed infants during different lactation stages. mSystems.

[80] Bych K., Mikš M.H., Johanson T., Hederos M.J., Vigsnæs L.K., Becker P. (2019). Production of HMOs using microbial hosts—From cell engineering to large scale production. Curr. Opin. Biotechnol..

[81] Vandenplas Y., Berger B., Carnielli V.P., Ksiazyk J., Lagström H., Sanchez Luna M., Migacheva N., Mosselmans J.M., Picaud J.C., Possner M. (2018). Human milk oligosaccharides: 2′-fucosyllactose (2′-FL) and lacto-*N*-*neo*tetraose (LN*n*T) in infant formula. Nutrients.

[82] Reverri E.J., Devitt A.A., Kajzer J.A., Baggs G.E., Borschel M.W. (2018). Review of the clinical experiences of feeding infants formula containing the human milk oligosaccharide 2′-fucosyllactose. Nutrients.

[83] Tannock G.W., Lawley B., Munro K., Gowri Pathmanathan S., Zhou S.J., Makrides M., Gibson R.A., Sullivan T., Prosser C.G., Lowry D. (2013). Comparison of the compositions of the stool microbiotas of infants fed goat milk formula, cow milk-based formula, or breast milk. Appl. Environ. Microbiol..

[84] Egan M., O’Connell Motherway M., Ventura M., van Sinderen D. (2014). Metabolism of sialic acid by *Bifidobacterium breve* UCC2003. Appl. Environ. Microbiol..

[85] Nishiyama K., Nagai A., Uribayashi K., Yamamoto Y., Mukai T., Okada N. (2018). Two extracellular sialidases from *Bifidobacterium bifidum* promote the degradation of sialyl-oligosaccharides and support the growth of *Bifidobacterium breve*. Anaerobe.

[86] Frese S.A., Hutton A.A., Contreras L.N., Shaw C.A., Palumbo M.C., Casaburi G., Xu G., Davis J.C.C., Lebrilla C.B., Henrick B.M. (2017). Persistence of supplemented *Bifidobacterium longum* subsp. infantis EVC001 in breastfed infants. mSphere.

[87] Underwood M.A., Davis J.C.C., Kalanetra K.M., Gehlot S., Patole S., Tancredi D.J., Mills D.A., Lebrilla C.B., Simmer K. (2017). Digestion of human milk oligosaccharides by *Bifidobacterium breve* in the premature infant. J. Pediatr. Gastroenterol. Nutr..

[88] Yu Z.T., Chen C., Newburg D.S. (2013). Utilization of major fucosylated and sialylated human milk oligosaccharides by isolated human gut microbes. Glycobiology.

[89] Bidart G.N., Rodriguez-Díaz J., Yebra M.J. (2016). The extracellular wall-bound β-*N*-acetylglucosaminidase from *Lactobacillus casei* is involved in the metabolism of the human milk oligosaccharide lacto-*N*-triose. Appl. Environ. Microbiol..

[90] Bidart G.N., Rodriguez-Díaz J., Monedero V., Yebra M.J. (2014). A unique gene cluster for the utilization of the mucosal and human milk-associated glycans galacto-*N*-biose and lacto-*N*-biose in *Lactobacillus casei*. Mol. Microbiol..

