# Supplementary material for: Varied Pathways of Infant Gut-Associated Bifidobacterium to Assimilate Human Milk Oligosaccharides: Prevalence of the Gene Set and Its Correlation with Bifidobacteria-Rich Microbiota Formation
Source: Nutrients. 2019 Dec 26;12(1):71. doi: 10.3390/nu12010071 (PMC7019425; doi:10.3390/nu12010071)
Supplement: Supplementary file 1 [file nutrients-12-00071-s001.zip › nutrients-12-00071-s001/nutrients-654423-supplementary material.pdf]

Supplementary Material for

Varied pathways of infant gut-associated *Bifidobacterium* to assimilate human milk oligosaccharides: prevalence of the gene set and its correlation with bifidobacteria-rich microbiota formation

Mikiyasu Sakanaka [1,2], Aina Gotoh [3], Keisuke Yoshida [4], Toshitaka Odamaki [4], Hiroka Koguchi [5], Jin-zhong Xiao [4], Motomitsu Kitaoka [6], and Takane Katayama [3,*]

[1] National Food Institute, Technical University of Denmark, Kemitorvet, DK-2800 Kgs. Lyngby, Denmark; miksak@dtu.dk (M.S.)
[2] Research Institute for Bioresources and Biotechnology, Ishikawa Prefectural University, Nonoichi, Ishikawa 921-8836, Japan
[3] Graduate school of Biostudies, Kyoto University, Kyoto 606-8502, Japan; gotoh.aina.44s@st.kyoto-u.ac.jp (A.G.).
[4] Next Generation Science Institute, Morinaga Milk Industry Co., Ltd., Zama, Kanagawa 252-8583, Japan; keisuke-yoshida826@morinagamilk.co.jp (K.Y.); t-odamak@morinagamilk.co.jp (T.O.); j_xiao@morinagamilk.co.jp (J.-z.X.)
[5] Department of Biotechnology and Bioengineering, Technical University of Denmark, Søltofts Plads, DK-2800 Kgs. Lyngby, Denmark; hirosa@dtu.dk (H.K.)
[6] The Faculty of Agriculture, Niigata University, Niigata 950-2181, Japan; mkitaoka@agr.niigata-u.ac.jp (M.K.)
* Correspondence: takane@lif.kyoto-u.ac.jp; Tel.: +81-75-753-9233.

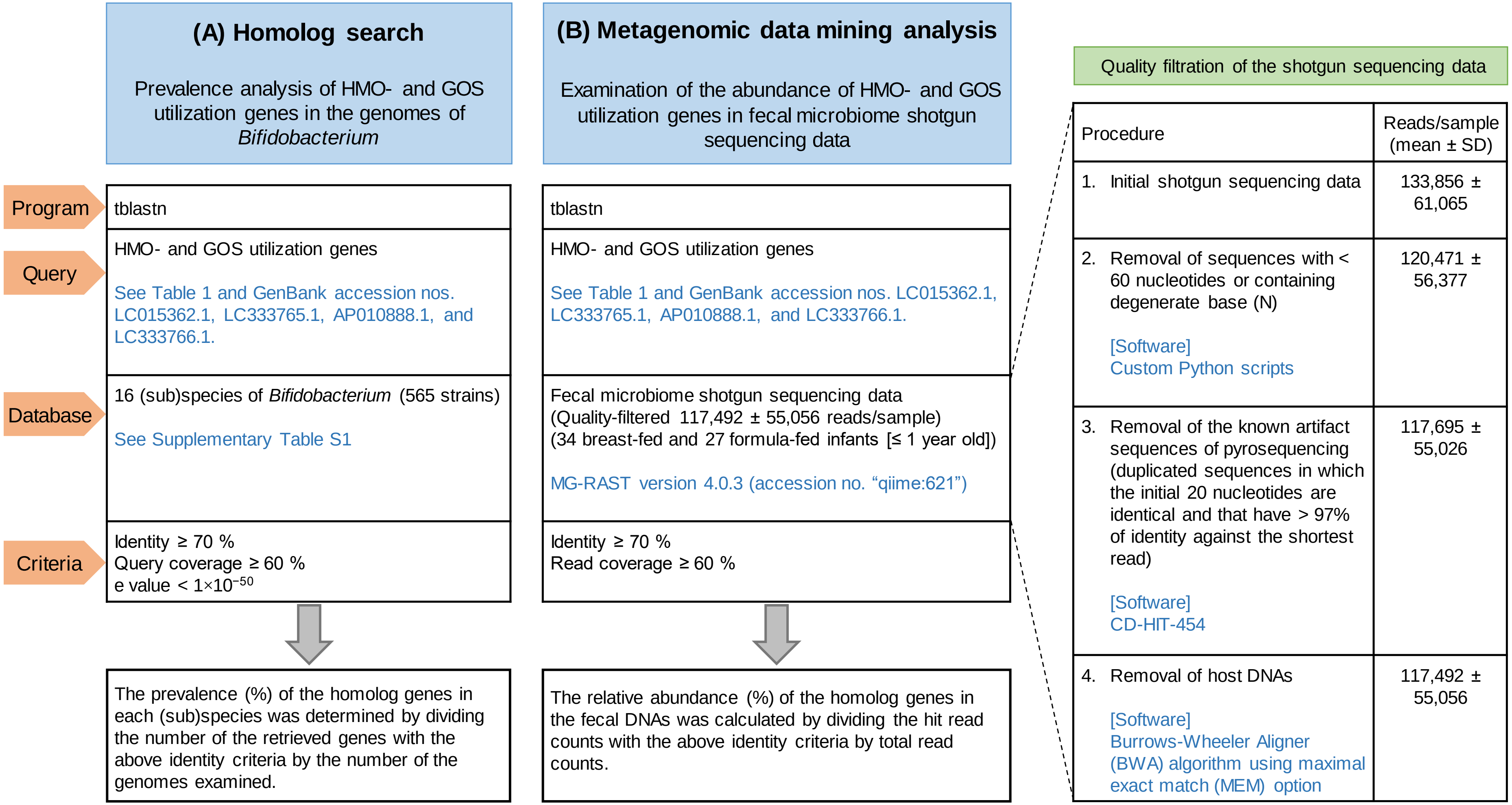

Figure S1. Graphical abstract of the *in silico* analysis procedures adopted in this study. (**A**) Prevalence analysis of HMO- and GOS utilization genes in the genomes of *Bifidobacterium* (homolog search). (**B**) Examination of the abundance of HMO- and GOS utilization genes in fecal microbiome shotgun sequencing data (metagenomic data mining analysis).

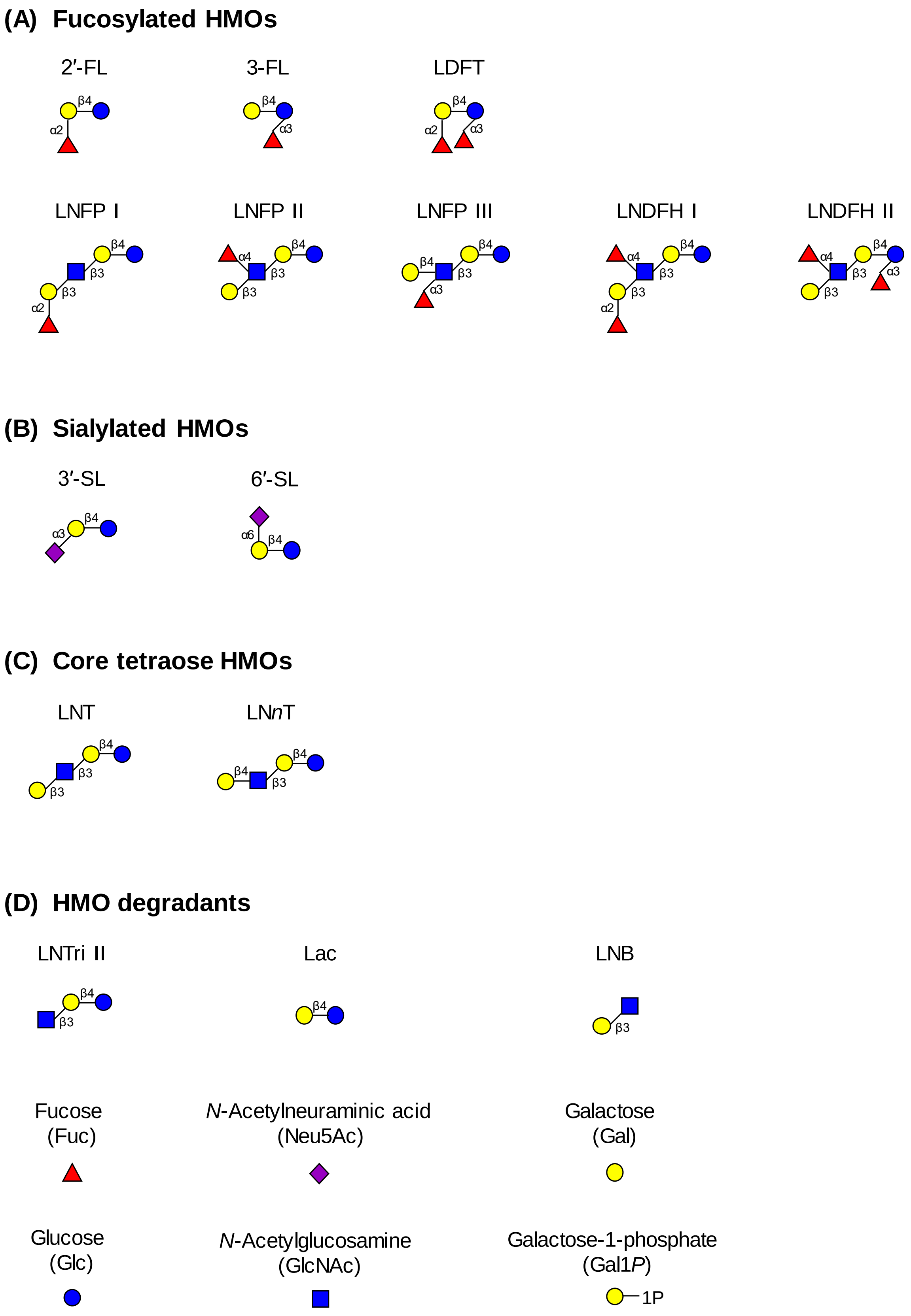

Figure S2. Representative twelve HMO molecules described in this study, and the HMO degradants produced in the assimilation pathways. (**A**) Fucosylated HMOs; (**B**) Sialylated HMOs; (**C**) Core tetraose HMOs; (**D**) HMO degradants.

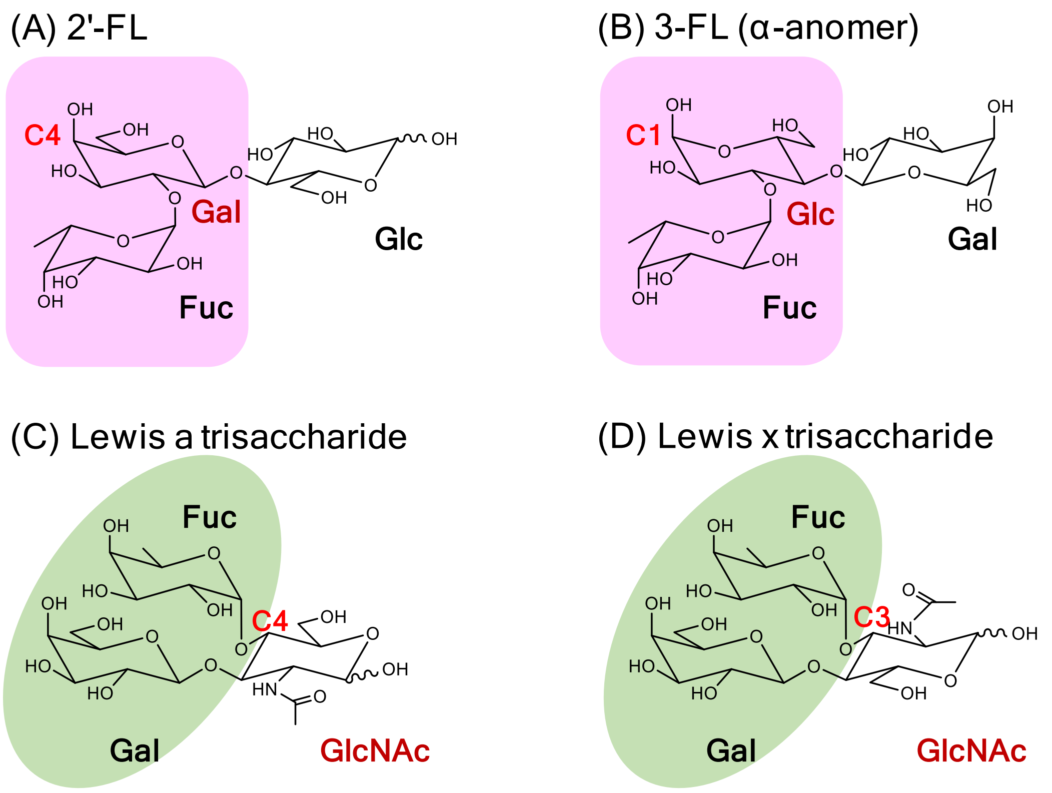

Figure S3. Unique specificity observed for α-L-fucosidases can be explained by the structural similarity between the different substrates. (**A, B**) Structural resemblance between 2'-FL and 3-FL in the Fuc-Gal/Glc moiety. Note that Glc residue of 3-FL adopts α-anomeric configuration. (**C, D**) The Lewis a/x trisaccharide structures share an identical steric motif at Gal and Fuc positions.

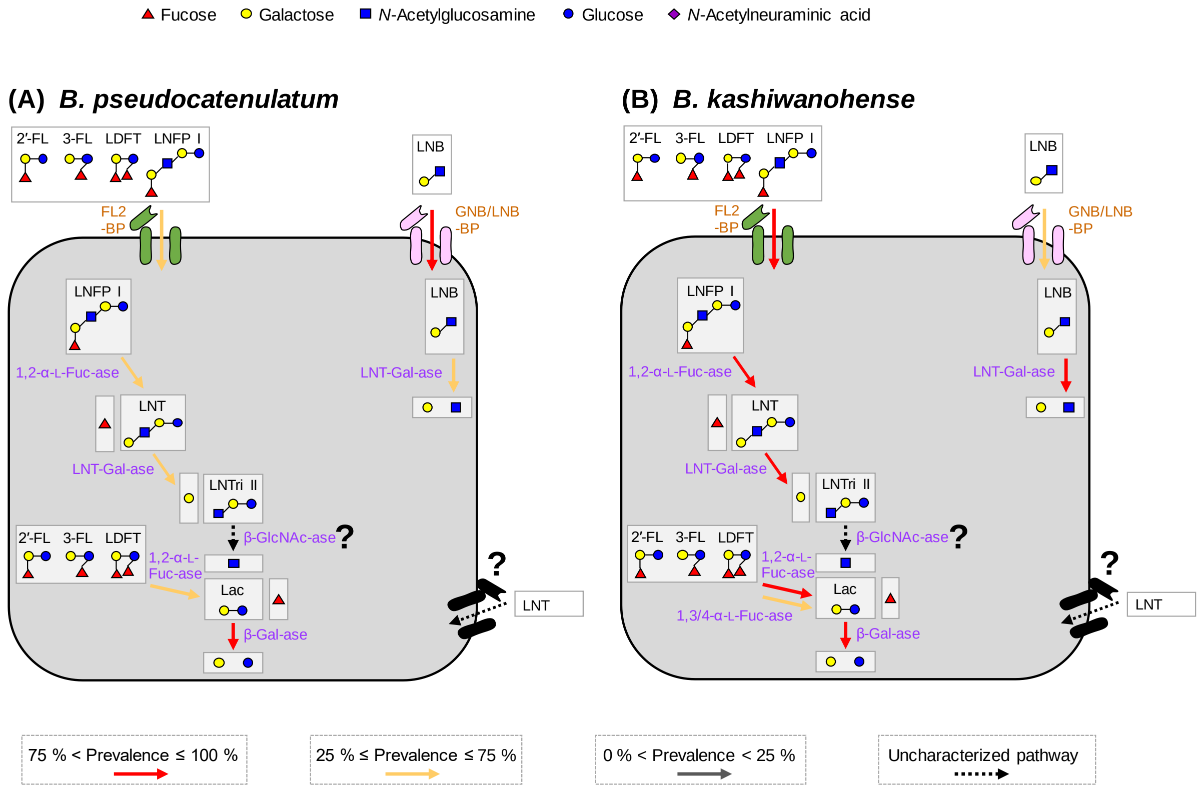

Figure S4. HMO utilization pathways of *B. pseudocatenulatum* (**A**) and *B. kashiwanohense* (**B**). Degradation pathways for the representative HMO molecules are shown. The arrows with different colors indicate the prevalence of respective homolog genes in each species (see Figure 2). Red: > 75 %; yellow, 25–75 %; and gray: < 25 %. The uncharacterized pathways are indicated by dotted black arrows. The transporter homologs are in brown letters, while intracellular enzyme homologs are in purple letters.

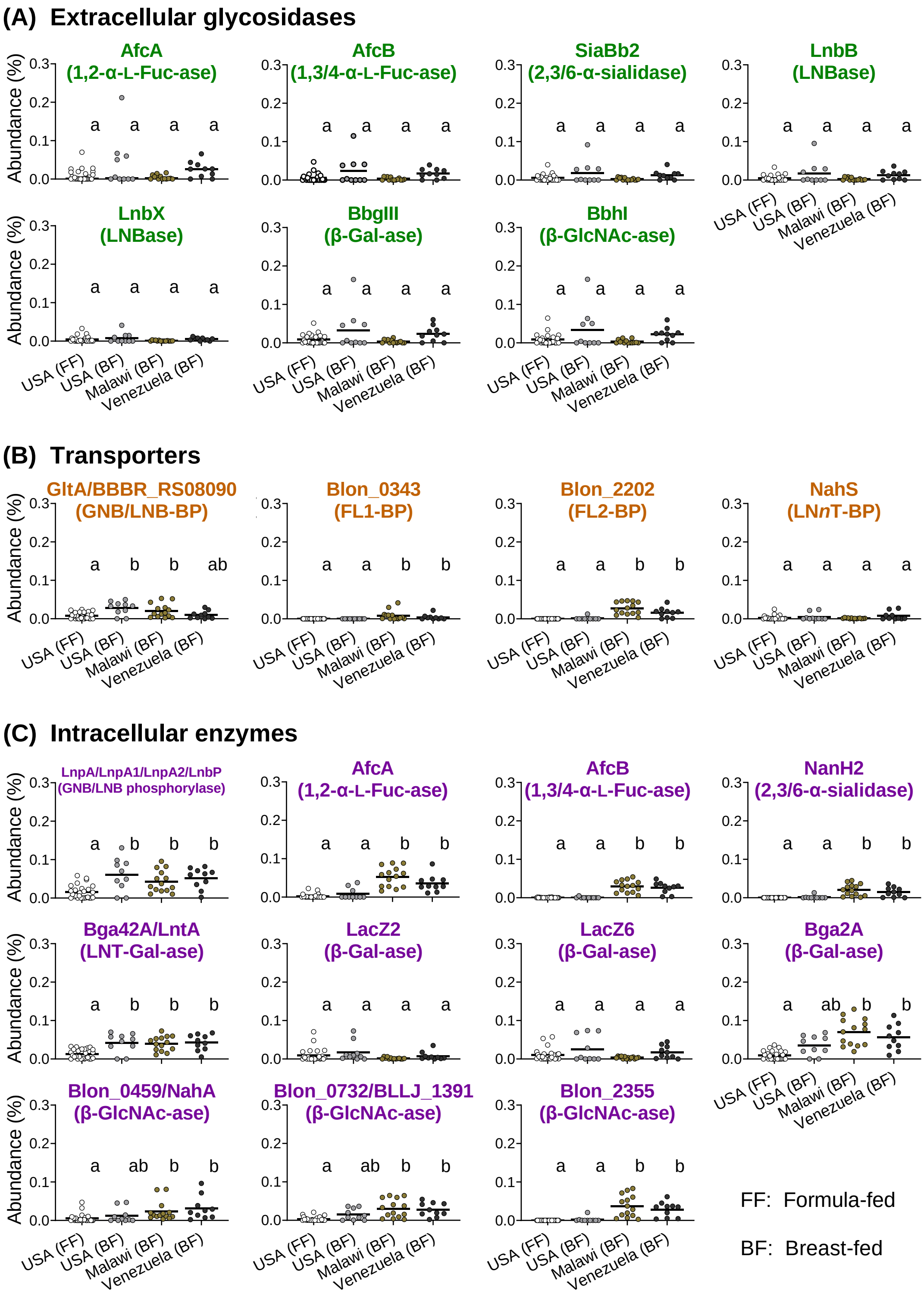

Figure S5. Metagenomic data mining analysis of HMO-related genes of bifidobacteria. (**A–C**) The abundances (%) of the genes encoding extracellular glycosidases (A), transporters (B), and intracellular enzymes (C) detected in the metagenomic data were compared between formula-fed infants living in USA (n = 27), breast-fed infants residing in USA (n = 10), Malawi (n = 14), and Venezuela (n = 10). Data from Yatsunenko et al. [39] (n = 27 for FF and n = 34 for BF; 117,492 ± 55,056 reads/sample) were used for the analysis. Different letters (a–c) indicate statistically significant differences among the four groups ($p < 0.05$, Dunne's test). BF: breast-fed; FF: formula-fed.

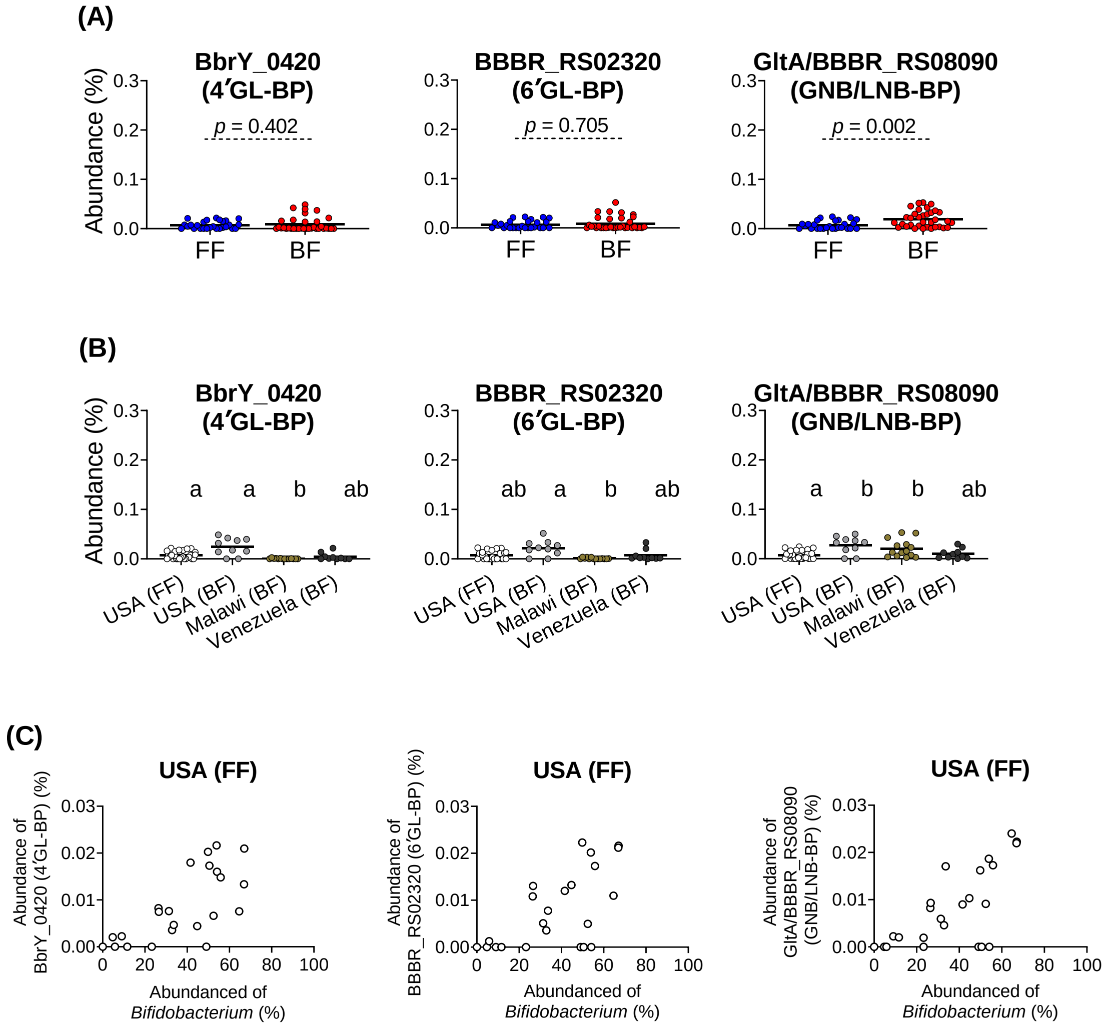

Figure S6. Metagenomic data mining analysis of GOS transporter homolog genes in bifidobacteria. (**A**) The abundances (%) of the genes (GenBank accession numbers of LC015362.1, LC333765.1, AP010888.1, and LC333766.1) detected in the metagenomic data were compared between formula-fed (n = 27) and breast-fed (n = 34) infants (≤ 1 year old), as in Figure 3. Mann-Whitney *U*-test was used to evaluate statistical significance. (**B**) The data in (A) were further divided into formula-fed infants living in USA (n = 27) and breast-fed infants residing in USA (n = 10), Malawi (n = 14), and Venezuela (n = 10). Different letters (a–c) indicate statistically significant differences among the four groups ($p < 0.05$, Dunne's test). (**C**) Correlation between relative abundances of GOS transporters and *Bifidobacterium* in formula-fed USA infants. Spearman's rank correlation coefficients are shown in Figure 4B. BF: breast-fed; FF: formula-fed; GL-BP: galactosyllactose-binding protein.

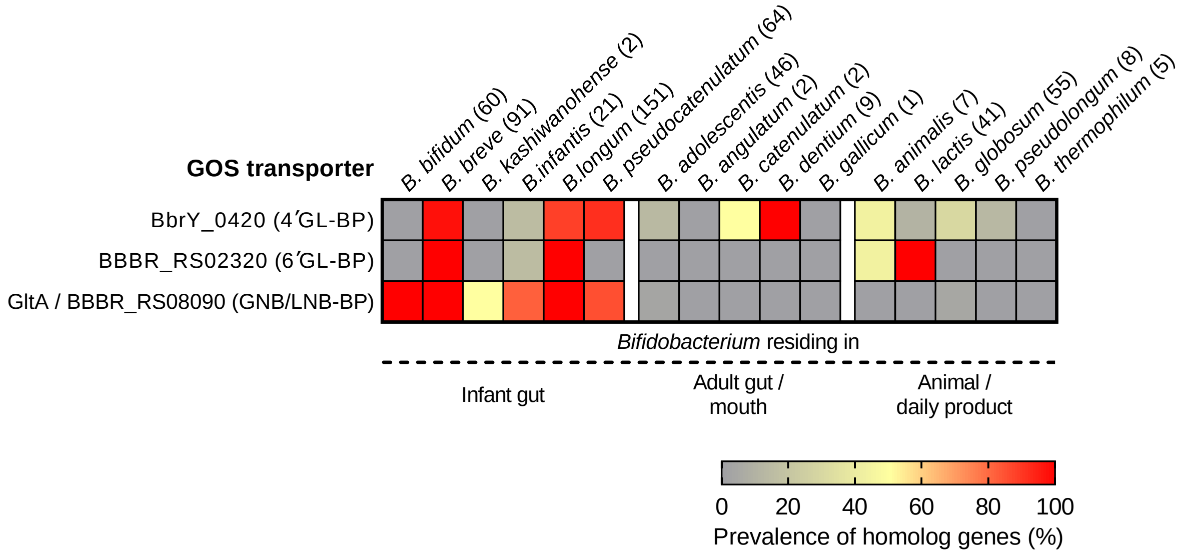

Figure S7. Prevalence of GOS transporter genes in the genomes of bifidobacteria. Occurrence of the homolog genes (identity ≥ 70 %, query coverage ≥ 60 %, e value < 1×10^{-50}) among the sixteen *Bifidobacterium* (sub)species was examined by tblastn analysis (BLAST+ v2.9.0). Amino acid sequences of the query genes were obtained from GenBank under the accession numbers of LC015362.1, LC333765.1, AP010888.1, and LC333766.1. The prevalence (%) in each species was determined by dividing the number of the retrieved hit genes with the above identity criterion by the number of the genomes examined (values in parentheses). The results are shown as a heatmap. GL-BP: galactosyllactose-binding protein.

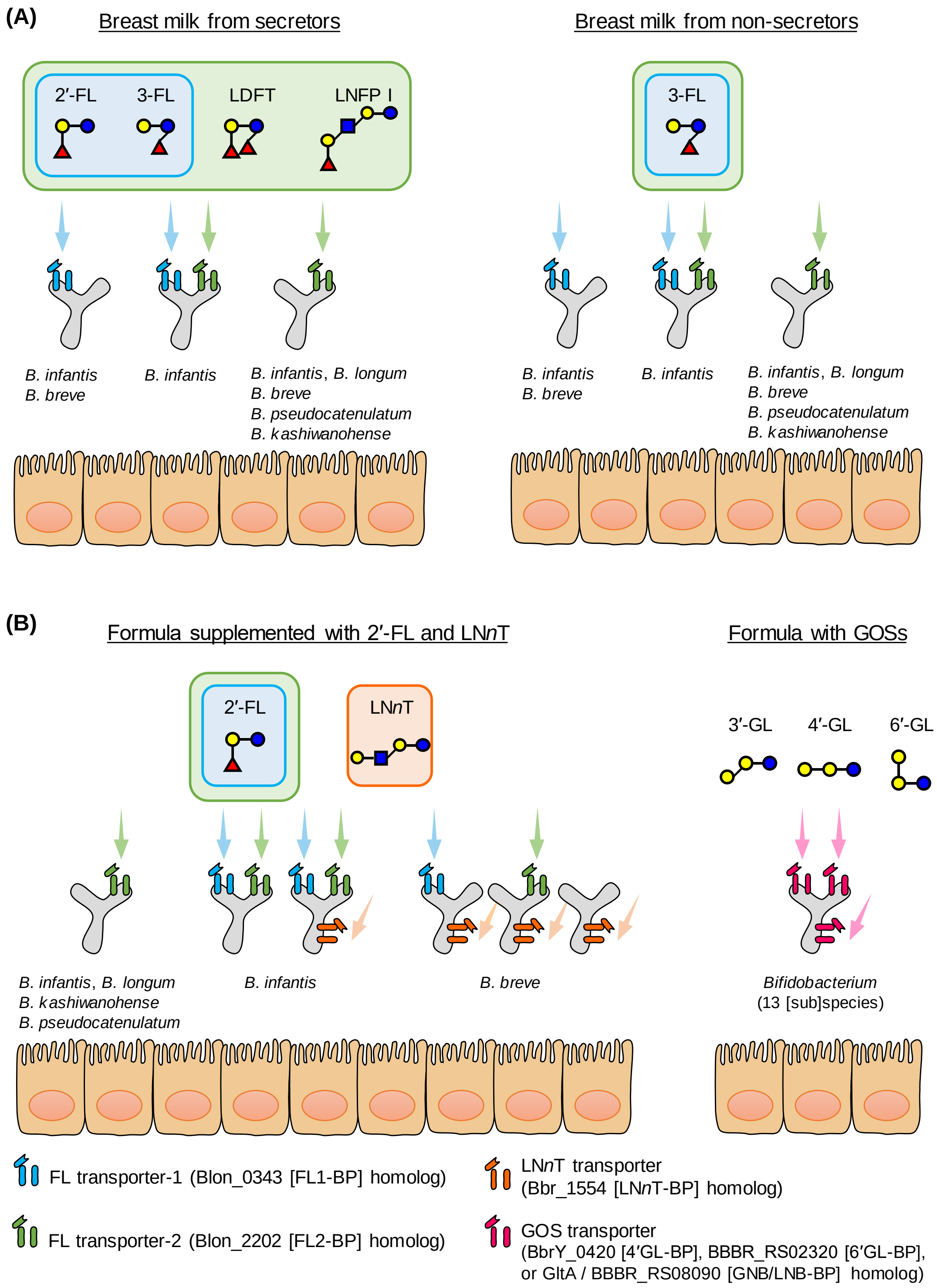

Figure S8. Bifidobacteria-rich microbiota formation processes in infant guts can differ between feeding strategies and be partially dependent on specific transporters. (**A**) *Bifidobacterium* expressing FL transporter-1 and -2 can consume several fucosylated HMOs that are abundantly present in secretors' (left panel) and non-secretors' (right panel) milk. (**B**) 2′-FL- and LN*n*T supplemented formula can promote the growth of *Bifidobacterium* specie/strains carrying the corresponding transporters, whereas formula with GOS supplementation stimulates the growth of bifidobacteria carrying GOS transporter(s). *Bifidobacterium* species that are assumed to proliferate under the different feeding conditions are shown based on the conservation profile of respective transporters (see Figure 2 and Supplementary Figure S7). FL transporter-1, FL transporter-2, LN*n*T transporter, and GOS transporters are shown in blue, green, orange, and pink, respectively. GL-BP: galactosyllactose-binding protein.
