# Supplementary material for: Varied Pathways of Infant Gut-Associated Bifidobacterium to Assimilate Human Milk Oligosaccharides: Prevalence of the Gene Set and Its Correlation with Bifidobacteria-Rich Microbiota Formation
Source: Nutrients. 2019 Dec 26;12(1):71. doi: 10.3390/nu12010071 (PMC7019425; doi:10.3390/nu12010071)
Supplement: Supplementary file 1 [file nutrients-12-00071-s001.zip › nutrients-12-00071-s001/nutrients-654423-supplementary Table S1.docx]

| **Table S1.** The accession numbers of *Bifidobacterium* strains used for the *in silico* analysis | |
| --- | --- |
| RefSeq assembly accession no. | *Bifidobacterium* strain |
| GCF_005845205.1 | *B. adolescentis* 1001271st1_A4 |
| GCF_003030905.1 | *B. adolescentis* 1-11 |
| GCF_001010915.1 | *B. adolescentis* 150 |
| GCF_002075965.1 | *B. adolescentis* 1892B |
| GCF_000737885.1 | *B. adolescentis* 22L |
| GCF_001406455.1 | *B. adolescentis* 2789STDY5608824 |
| GCF_001406735.1 | *B. adolescentis* 2789STDY5608862 |
| GCF_001406215.1 | *B. adolescentis* 2789STDY5834850 |
| GCF_002107925.1 | *B. adolescentis* 42B |
| GCF_002108015.1 | *B. adolescentis* 487B |
| GCF_003429385.1 | *B. adolescentis* 6 |
| GCF_002108035.1 | *B. adolescentis* 703B |
| GCF_002107955.1 | *B. adolescentis* 70B |
| GCF_002108045.1 | *B. adolescentis* AD2-8 |
| GCF_003465205.1 | *B. adolescentis* AF14-56 |
| GCF_003464325.1 | *B. adolescentis* AF15-3 |
| GCF_003458805.1 | *B. adolescentis* AF21-27 |
| GCF_003457765.1 | *B. adolescentis* AF28-4AC |
| GCF_002107975.1 | *B. adolescentis* AL12-4 |
| GCF_002108075.1 | *B. adolescentis* AL46-2 |
| GCF_002108095.1 | *B. adolescentis* AL46-7 |
| GCF_003472265.1 | *B. adolescentis* AM12-20 |
| GCF_003472245.1 | *B. adolescentis* AM12-59 |
| GCF_003473105.1 | *B. adolescentis* AM13-11 |
| GCF_003472095.1 | *B. adolescentis* AM14-37 |
| GCF_003469145.1 | *B. adolescentis* AM34-11 |
| GCF_003468385.1 | *B. adolescentis* AM36-3AC |
| GCF_003467335.1 | *B. adolescentis* AM41-17 |
| GCF_000010425.1 | *B. adolescentis* ATCC 15703 |
| GCF_000817995.1 | *B. adolescentis* BBMN23 |
| GCF_004167585.1 | *B. adolescentis* ca_0067 |
| GCF_000829865.1 | *B. adolescentis* IVS-1 |
| GCF_001756865.1 | *B. adolescentis* Km 4 |
| GCF_000154085.1 | *B. adolescentis* L2-32 |
| GCF_002108135.1 | *B. adolescentis* LMG 10733 |
| GCF_002107995.1 | *B. adolescentis* LMG 10734 |
| GCF_002108155.1 | *B. adolescentis* LMG 11579 |
| GCF_002108165.1 | *B. adolescentis* LMG 18897 |
| GCF_003462885.1 | *B. adolescentis* OF04-5 |
| GCF_003462895.1 | *B. adolescentis* OF04-9AC |
| GCF_003856735.1 | *B. adolescentis* P2P3 |
| GCF_003437775.1 | *B. adolescentis* TF06-10AC |
| GCF_003437755.1 | *B. adolescentis* TF06-29 |
| GCF_003437735.1 | *B. adolescentis* TF06-2AC |
| GCF_003436185.1 | *B. adolescentis* TM06-4 |
| GCF_003466335.1 | *B. adolescentis* TM06-51 |
| GCF_000966445.2 | *B. angulatum* GT102 |
| GCF_001025155.1 | *B. angulatum* JCM 7096 |
| GCF_004154625.1 | *B. animalis* subsp. *animalis* 2006B |
| GCF_000260715.1 | *B. animalis* subsp. *animalis* ATCC 25527 |
| GCF_001264055.1 | *B. animalis* subsp. *animalis* ATCC 27672 |
| GCF_003671995.1 | *B. animalis* subsp. *animalis* CNCM I-4602 |
| GCF_001039715.1 | *B. animalis* subsp. *animalis* IM386 |
| GCF_001263835.1 | *B. animalis* subsp. *animalis* MCC 1489 |
| GCF_001688645.2 | *B. animalis* subsp. *animalis* YL2 |
| GCF_004154565.1 | *B. animalis* subsp. *lactis* 1316B |
| GCF_004154695.1 | *B. animalis* subsp. *lactis* 1395B |
| GCF_004154555.1 | *B. animalis* subsp. *lactis* 1528B |
| GCF_004154655.1 | *B. animalis* subsp. *lactis* 1802B |
| GCF_004154545.1 | *B. animalis* subsp. *lactis* 1808B |
| GCF_004154535.1 | *B. animalis* subsp. *lactis* 1811B |
| GCF_004154525.1 | *B. animalis* subsp. *lactis* 1813B |
| GCF_004154435.1 | *B. animalis* subsp. *lactis* 1821B |
| GCF_004154475.1 | *B. animalis* subsp. *lactis* 1843B |
| GCF_004154445.1 | *B. animalis* subsp. *lactis* 1869B |
| GCF_004154645.1 | *B. animalis* subsp. *lactis* 2007B |
| GCF_004154425.1 | *B. animalis* subsp. *lactis* 2010B |
| GCF_004154455.1 | *B. animalis* subsp. *lactis* 2011B |
| GCF_001892925.1 | *B. animalis* subsp. *lactis* 646 |
| GCF_000021425.1 | *B. animalis* subsp. *lactis* AD011 |
| GCF_001263975.1 | *B. animalis* subsp. *lactis* ATCC 27536 |
| GCF_000471945.1 | *B. animalis* subsp. *lactis* ATCC 27673 |
| GCF_001263985.1 | *B. animalis* subsp. *lactis* ATCC 27674 |
| GCF_000277325.1 | *B. animalis* subsp. *lactis* B420 |
| GCF_000025245.1 | *B. animalis* subsp. *lactis* BB-12 |
| GCF_000818055.1 | *B. animalis* subsp. *lactis* BF052 |
| GCF_000277345.1 | *B. animalis* subsp. *lactis* Bi-07 |
| GCF_000022705.1 | *B. animalis* subsp. *lactis* Bl-04; ATCC SD5219 |
| GCF_000414215.1 | *B. animalis* subsp. *lactis* Bl12 |
| GCF_000224965.2 | *B. animalis* subsp. *lactis* BLC1 |
| GCF_000240765.1 | *B. animalis* subsp. *lactis* BS 01 |
| GCF_000612705.1 | *B. animalis* subsp. *lactis* CECT 8145 |
| GCF_003094915.1 | *B. animalis* subsp. *lactis* CF3_2 |
| GCF_000220885.1 | *B. animalis* subsp. *lactis* CNCM I-2494 |
| GCF_003094835.1 | *B. animalis* subsp. *lactis* DS23_2 |
| GCF_003094815.1 | *B. animalis* subsp. *lactis* DS24_2 |
| GCF_003094775.1 | *B. animalis* subsp. *lactis* DS27_2 |
| GCF_003095015.1 | *B. animalis* subsp. *lactis* DS28_2 |
| GCF_003606305.1 | *B. animalis* subsp. *lactis* HN019 |
| GCF_003428375.1 | *B. animalis* subsp. *lactis* IDCC4301 |
| GCF_000816205.1 | *B. animalis* subsp. *lactis* KLDS2.0603 |
| GCF_002914815.1 | *B. animalis* subsp. *lactis* LMG P-17502_1 |
| GCF_002914895.1 | *B. animalis* subsp. *lactis* LMG P-17502_2 |
| GCF_003390755.1 | *B. animalis* subsp. *lactis* S7 |
| GCF_003970855.1 | *B. animalis* subsp. *lactis* UBBLa 70 |
| GCF_000092765.1 | *B. animalis* subsp. *lactis* V9 |
| GCF_002469385.1 | *B. bifidum* 1 G1971 |
| GCF_005844365.1 | *B. bifidum* 1001271st1_H11 |
| GCF_001020255.1 | *B. bifidum* 156B |
| GCF_002076105.1 | *B. bifidum* 1887B |
| GCF_001405355.1 | *B. bifidum* 2789STDY5608877 |
| GCF_001020375.1 | *B. bifidum* 324B |
| GCF_001595435.1 | *B. bifidum* 791 |
| GCF_001020355.1 | *B. bifidum* 85B |
| GCF_001020245.1 | *B. bifidum* A8 |
| GCF_003465425.1 | *B. bifidum* AF11-25B |
| GCF_003474045.1 | *B. bifidum* AF45-10 |
| GCF_003472345.1 | *B. bifidum* AM12-10 |
| GCF_003473145.1 | *B. bifidum* AM12-9 |
| GCF_003471595.1 | *B. bifidum* AM18-1 |
| GCF_003471555.1 | *B. bifidum* AM18-11 |
| GCF_003470615.1 | *B. bifidum* AM18-12AC |
| GCF_003470585.1 | *B. bifidum* AM18-29 |
| GCF_003468415.1 | *B. bifidum* AM36-1AC |
| GCF_003467985.1 | *B. bifidum* AM42-15AC |
| GCF_001281345.1 | *B. bifidum* BF3 |
| GCF_000265095.1 | *B. bifidum* BGN4 |
| GCF_001685685.1 | *B. bifidum* BI-14 |
| GCF_005844205.1 | *B. bifidum* BSD2780061688st1_G1 |
| GCF_004167365.1 | *B. bifidum* ca_0067 |
| GCF_900112465.1 | *B. bifidum* Calf96 |
| GCF_003094795.1 | *B. bifidum* DS25_23 |
| GCF_003095035.1 | *B. bifidum* DS26_23 |
| GCF_003094755.1 | *B. bifidum* DS30_23 |
| GCF_003094735.1 | *B. bifidum* DS31_23 |
| GCF_003094715.1 | *B. bifidum* DS32_23 |
| GCF_003094645.1 | *B. bifidum* DS33_23 |
| GCF_004799295.1 | *B. bifidum* ICIS-202 |
| GCF_002114145.1 | *B. bifidum* ICIS-310 |
| GCF_003790385.1 | *B. bifidum* ICIS-643 |
| GCF_001020405.1 | *B. bifidum* IPLA 20015 |
| GCF_001020265.1 | *B. bifidum* IPLA 20017 |
| GCF_001025135.1 | *B. bifidum* JCM 1255 |
| GCF_001020275.1 | *B. bifidum* LMG 11582 |
| GCF_001020335.1 | *B. bifidum* LMG 11583 |
| GCF_001020415.1 | *B. bifidum* LMG 13195 |
| GCF_001020325.1 | *B. bifidum* LMG 13200 |
| GCF_001546225.1 | *B. bifidum* MJR8628B |
| GCF_000155395.1 | *B. bifidum* NCIMB 41171 |
| GCF_002845845.1 | *B. bifidum* PRI 1 |
| GCF_000165905.1 | *B. bifidum* PRL2010 |
| GCF_000164965.1 | *B. bifidum* S17 |
| GCF_003390735.1 | *B. bifidum* S6 |
| GCF_003437945.1 | *B. bifidum* TF05-1 |
| GCF_003437785.1 | *B. bifidum* TF05-39 |
| GCF_003437345.1 | *B. bifidum* TF06-13 |
| GCF_003437325.1 | *B. bifidum* TF06-14AC |
| GCF_003437175.1 | *B. bifidum* TF07-22 |
| GCF_003436655.1 | *B. bifidum* TM02-15 |
| GCF_003436635.1 | *B. bifidum* TM02-17 |
| GCF_003436575.1 | *B. bifidum* TM04-12 |
| GCF_003466485.1 | *B. bifidum* TM05-15 |
| GCF_003466395.1 | *B. bifidum* TM06-10 |
| GCF_003466365.1 | *B. bifidum* TM06-5 |
| GCF_003436135.1 | *B. bifidum* TM07-4AC |
| GCF_003573895.1 | *B. bifidum* TMC 3115 |
| GCF_002838465.1 | *B. breve* 017W439 |
| GCF_002838545.1 | *B. breve* 082W48 |
| GCF_000568955.1 | *B. breve* 12L |
| GCF_002838565.1 | *B. breve* 139W423 |
| GCF_002838525.1 | *B. breve* 180W83 |
| GCF_002076075.1 | *B. breve* 1889B |
| GCF_002076055.1 | *B. breve* 1891B |
| GCF_002075865.1 | *B. breve* 1900B |
| GCF_002838485.1 | *B. breve* 215W447a |
| GCF_000568895.1 | *B. breve* 2L |
| GCF_003370195.1 | *B. breve* 30-1 |
| GCF_000568875.1 | *B. breve* 31L |
| GCF_003370175.1 | *B. breve* 322-1 |
| GCF_003370155.1 | *B. breve* 43803 |
| GCF_000569055.1 | *B. breve* 689b |
| GCF_002271275.1 | *B. breve* 7E |
| GCF_003370295.1 | *B. breve* 91-1 |
| GCF_000213865.1 | *B. breve* ACS-071-V-Sch8b |
| GCF_001189355.1 | *B. breve* BBRI4 |
| GCF_900157125.1 | *B. breve* Bifido_07 |
| GCF_900157105.1 | *B. breve* Bifido_10 |
| GCF_004319685.1 | *B. breve* BR03 |
| GCF_001685705.1 | *B. breve* BR-06 |
| GCF_001685725.1 | *B. breve* BR-07 |
| GCF_001685745.1 | *B. breve* BR-10 |
| GCF_001685765.1 | *B. breve* BR-14 |
| GCF_001685785.1 | *B. breve* BR-15 |
| GCF_001685805.1 | *B. breve* BR-19 |
| GCF_001685825.1 | *B. breve* BR-20 |
| GCF_001685845.1 | *B. breve* BR-21 |
| GCF_001281425.1 | *B. breve* BR3 |
| GCF_001685865.1 | *B. breve* BR-A29 |
| GCF_001685885.1 | *B. breve* BR-C29 |
| GCF_001685905.1 | *B. breve* BR-H29 |
| GCF_001685925.1 | *B. breve* BR-I29 |
| GCF_001685945.1 | *B. breve* BR-L29 |
| GCF_000247755.1 | *B. breve* CECT 7263 |
| GCF_002838585.1 | *B. breve* CNCM I-4321 |
| GCF_000226175.1 | *B. breve* DPC 6330 |
| GCF_002838225.1 | *B. breve* DRBB26 |
| GCF_002838445.1 | *B. breve* DRBB27 |
| GCF_002838505.1 | *B. breve* DRBB28 |
| GCF_002838705.1 | *B. breve* DRBB29 |
| GCF_002838725.1 | *B. breve* DRBB30 |
| GCF_003095095.1 | *B. breve* DS15_17 |
| GCF_003813065.1 | *B. breve* FDAARGOS_561 |
| GCF_001546235.1 | *B. breve* GED8481 |
| GCF_000411435.1 | *B. breve* HPH0326 |
| GCF_001025175.1 | *B. breve* JCM 1192 |
| GCF_000568975.1 | *B. breve* JCM 7017 |
| GCF_000569015.1 | *B. breve* JCM 7019 |
| GCF_000466545.1 | *B. breve* JCP7499 |
| GCF_001990225.1 | *B. breve* LMC520 |
| GCF_002914865.1 | *B. breve* LMG S-29190 |
| GCF_003860285.1 | *B. breve* lw01 |
| GCF_001264045.1 | *B. breve* MCC 0121 |
| GCF_001264035.1 | *B. breve* MCC 0305 |
| GCF_001263845.1 | *B. breve* MCC 0476 |
| GCF_001264095.1 | *B. breve* MCC 1094 |
| GCF_001263855.1 | *B. breve* MCC 1114 |
| GCF_001263915.1 | *B. breve* MCC 1128 |
| GCF_001264105.1 | *B. breve* MCC 1340 |
| GCF_001264135.1 | *B. breve* MCC 1454 |
| GCF_001264155.1 | *B. breve* MCC 1604 |
| GCF_001263935.1 | *B. breve* MCC 1605 |
| GCF_003370105.1 | *B. breve* N6D12 |
| GCF_000569035.1 | *B. breve* NCFB 2258 |
| GCF_002838245.1 | *B. breve* NRBB01 |
| GCF_002838265.1 | *B. breve* NRBB02 |
| GCF_002838285.1 | *B. breve* NRBB04 |
| GCF_002838745.1 | *B. breve* NRBB08 |
| GCF_002838325.1 | *B. breve* NRBB09 |
| GCF_002838305.1 | *B. breve* NRBB11 |
| GCF_002838605.1 | *B. breve* NRBB18 |
| GCF_002838625.1 | *B. breve* NRBB19 |
| GCF_002838645.1 | *B. breve* NRBB20 |
| GCF_002838665.1 | *B. breve* NRBB27 |
| GCF_002838685.1 | *B. breve* NRBB49 |
| GCF_002838365.1 | *B. breve* NRBB50 |
| GCF_002838405.1 | *B. breve* NRBB51 |
| GCF_002838385.1 | *B. breve* NRBB52 |
| GCF_002838425.1 | *B. breve* NRBB56 |
| GCF_002838345.1 | *B. breve* NRBB57 |
| GCF_900102865.1 | *B. breve* RP2 |
| GCF_000569075.1 | *B. breve* S27 |
| GCF_004802595.1 | *B. breve* UBBR-01 |
| GCF_000220135.1 | *B. breve* UCC2003 |
| GCF_002871815.1 | *B. breve* UMB0089 |
| GCF_002861455.1 | *B. breve* UMB0915 |
| GCF_003370125.1 | *B. breve* W20-13 |
| GCF_003370265.1 | *B. breve* W56 |
| GCF_002075855.1 | *B. catenulatum* 1899B |
| GCF_001025195.1 | *B. catenulatum* JCM 1194 |
| GCF_002075955.1 | *B. dentium* 1893B |
| GCF_004683745.1 | *B. dentium* ATCC 15424 |
| GCF_000172135.1 | *B. dentium* ATCC 27678 |
| GCF_000146775.1 | *B. dentium* ATCC 27679 |
| GCF_000024445.1 | *B. dentium* Bd1 |
| GCF_004167735.1 | *B. dentium* cx_0004 |
| GCF_001686085.1 | *B. dentium* DE-29 |
| GCF_001042595.1 | *B. dentium* JCM 1195 |
| GCF_000149165.1 | *B. dentium* JCVIHMP022 |
| GCF_000741205.1 | *B. gallicum* LMG 11596 |
| GCF_001042615.1 | *B. kashiwanohense* JCM 15439 |
| GCF_000800455.1 | *B. kashiwanohense* PV20-2 |
| GCF_000196575.1 | *B. longum* subsp. *infantis* 157F |
| GCF_002076025.1 | *B. longum* subsp. *infantis* 1888B |
| GCF_000020425.1 | *B. longum* subsp. *infantis* ATCC 15697 |
| GCF_004919065.1 | *B. longum* subsp. *infantis* Bi-26 |
| GCF_000825105.1 | *B. longum* subsp. *infantis* BIB1401242951 |
| GCF_000825125.1 | *B. longum* subsp. *infantis* BIB1401272845a |
| GCF_000825145.1 | *B. longum* subsp. *infantis* BIB1401272845b |
| GCF_000825005.1 | *B. longum* subsp. *infantis* BIC1206122787 |
| GCF_000825025.1 | *B. longum* subsp. *infantis* BIC1307292462 |
| GCF_000825045.1 | *B. longum* subsp. *infantis* BIC1401111250 |
| GCF_000825065.1 | *B. longum* subsp. *infantis* BIC1401212621a |
| GCF_000825085.1 | *B. longum* subsp. *infantis* BIC1401212621b |
| GCF_001281305.1 | *B. longum* subsp. *infantis* BT1 |
| GCF_000155415.1 | *B. longum* subsp. *infantis* CCUG 52486 |
| GCF_001051015.2 | *B. longum* subsp. *infantis* CECT 7210 |
| GCF_000730125.1 | *B. longum* subsp. *infantis* EK3 |
| GCF_001686105.1 | *B. longum* subsp. *infantis* IN-07 |
| GCF_001686125.1 | *B. longum* subsp. *infantis* IN-F29 |
| GCF_900445755.1 | *B. longum* subsp. *infantis* NCTC 13219 |
| GCF_001870755.1 | *B. longum* subsp. *infantis* TPY12-1 |
| GCF_004803425.1 | *B. longum* subsp. *infantis* UBBI-01 |
| GCF_000730105.1 | *B. longum* subsp. *longum* 1-5B |
| GCF_000261245.1 | *B. longum* subsp. *longum* 1-6B |
| GCF_000730035.1 | *B. longum* subsp. *longum* 17-1B |
| GCF_000261205.1 | *B. longum* subsp. *longum* 2-2B |
| GCF_000261225.1 | *B. longum* subsp. *longum* 35B |
| GCF_000261265.1 | *B. longum* subsp. *longum* 44B |
| GCF_001447975.1 | *B. longum* subsp. *longum* 7 |
| GCF_000730055.1 | *B. longum* subsp. *longum* 7-1B |
| GCF_000730045.1 | *B. longum* subsp. *longum* 72B |
| GCF_001447955.1 | *B. longum* subsp. *longum* 9 |
| GCF_001725985.1 | *B. longum* subsp. *longum* AH1206 |
| GCF_000003135.1 | *B. longum* subsp. *longum* ATCC 55813 |
| GCF_000166315.1 | *B. longum* subsp. *longum* BBMN68 |
| GCF_001275745.1 | *B. longum* subsp. *longum* BLOI2 |
| GCF_003342655.1 | *B. longum* subsp. *longum* BORI |
| GCF_004324325.1 | *B. longum* subsp. *longum* C11A10B |
| GCF_001446275.1 | *B. longum* subsp. *longum* CCUG30698 |
| GCF_001050555.1 | *B. longum* subsp. *longum* CECT 7347 |
| GCF_000410595.1 | *B. longum* subsp. *longum* CMCC P0001 |
| GCF_003094995.1 | *B. longum* subsp. *longum* DS32_3 |
| GCF_000730135.1 | *B. longum* subsp. *longum* EK13 |
| GCF_000730025.1 | *B. longum* subsp. *longum* EK5 |
| GCF_000210755.1 | *B. longum* subsp. *longum* F8 |
| GCF_000772485.1 | *B. longum* subsp. *longum* GT15 |
| GCF_000196555.1 | *B. longum* subsp. *longum* JCM 1217 |
| GCF_000092325.1 | *B. longum* subsp. *longum* JDM301 |
| GCF_000219455.1 | *B. longum* subsp. *longum* KACC 91563 |
| GCF_001686145.1 | *B. longum* subsp. *longum* LO-06 |
| GCF_001686165.1 | *B. longum* subsp. *longum* LO-10 |
| GCF_001686185.1 | *B. longum* subsp. *longum* LO-21 |
| GCF_001686205.1 | *B. longum* subsp. *longum* LO-C29 |
| GCF_001686225.1 | *B. longum* subsp. *longum* LO-K29a |
| GCF_001686245.1 | *B. longum* subsp. *longum* LO-K29b |
| GCF_001516925.1 | *B. longum* subsp. *longum* MC-42 |
| GCF_004334545.1 | *B. longum* subsp. *longum* MCC10002 |
| GCF_004334075.1 | *B. longum* subsp. *longum* MCC10003 |
| GCF_004334065.1 | *B. longum* subsp. *longum* MCC10004 |
| GCF_004334535.1 | *B. longum* subsp. *longum* MCC10006 |
| GCF_004334045.1 | *B. longum* subsp. *longum* MCC10007 |
| GCF_004334035.1 | *B. longum* subsp. *longum* MCC10008 |
| GCF_004334515.1 | *B. longum* subsp. *longum* MCC10009 |
| GCF_004334005.1 | *B. longum* subsp. *longum* MCC10010 |
| GCF_004334485.1 | *B. longum* subsp. *longum* MCC10011 |
| GCF_004333995.1 | *B. longum* subsp. *longum* MCC10012 |
| GCF_004333975.1 | *B. longum* subsp. *longum* MCC10014 |
| GCF_004333925.1 | *B. longum* subsp. *longum* MCC10015 |
| GCF_004334465.1 | *B. longum* subsp. *longum* MCC10016 |
| GCF_004333935.1 | *B. longum* subsp. *longum* MCC10017 |
| GCF_004333905.1 | *B. longum* subsp. *longum* MCC10018 |
| GCF_004334435.1 | *B. longum* subsp. *longum* MCC10019 |
| GCF_004333875.1 | *B. longum* subsp. *longum* MCC10021 |
| GCF_004333895.1 | *B. longum* subsp. *longum* MCC10022 |
| GCF_004333855.1 | *B. longum* subsp. *longum* MCC10023 |
| GCF_004333845.1 | *B. longum* subsp. *longum* MCC10025 |
| GCF_004334445.1 | *B. longum* subsp. *longum* MCC10027 |
| GCF_004334425.1 | *B. longum* subsp. *longum* MCC10028 |
| GCF_004333795.1 | *B. longum* subsp. *longum* MCC10029 |
| GCF_004334355.1 | *B. longum* subsp. *longum* MCC10030 |
| GCF_004333785.1 | *B. longum* subsp. *longum* MCC10031 |
| GCF_004333775.1 | *B. longum* subsp. *longum* MCC10033 |
| GCF_004333765.1 | *B. longum* subsp. *longum* MCC10034 |
| GCF_004333715.1 | *B. longum* subsp. *longum* MCC10035 |
| GCF_004333735.1 | *B. longum* subsp. *longum* MCC10036 |
| GCF_004334365.1 | *B. longum* subsp. *longum* MCC10038 |
| GCF_004334335.1 | *B. longum* subsp. *longum* MCC10039 |
| GCF_004334345.1 | *B. longum* subsp. *longum* MCC10040 |
| GCF_004333695.1 | *B. longum* subsp. *longum* MCC10041 |
| GCF_004333675.1 | *B. longum* subsp. *longum* MCC10042 |
| GCF_004333635.1 | *B. longum* subsp. *longum* MCC10043 |
| GCF_004333645.1 | *B. longum* subsp. *longum* MCC10044 |
| GCF_004333625.1 | *B. longum* subsp. *longum* MCC10045 |
| GCF_004333575.1 | *B. longum* subsp. *longum* MCC10046 |
| GCF_004334325.1 | *B. longum* subsp. *longum* MCC10047 |
| GCF_004333565.1 | *B. longum* subsp. *longum* MCC10048 |
| GCF_004333555.1 | *B. longum* subsp. *longum* MCC10050 |
| GCF_004334285.1 | *B. longum* subsp. *longum* MCC10051 |
| GCF_004333535.1 | *B. longum* subsp. *longum* MCC10052 |
| GCF_004333515.1 | *B. longum* subsp. *longum* MCC10053 |
| GCF_004334245.1 | *B. longum* subsp. *longum* MCC10054 |
| GCF_004333455.1 | *B. longum* subsp. *longum* MCC10055 |
| GCF_004333475.1 | *B. longum* subsp. *longum* MCC10056 |
| GCF_004334255.1 | *B. longum* subsp. *longum* MCC10057 |
| GCF_004334215.1 | *B. longum* subsp. *longum* MCC10058 |
| GCF_004334235.1 | *B. longum* subsp. *longum* MCC10059 |
| GCF_004333465.1 | *B. longum* subsp. *longum* MCC10060 |
| GCF_004333445.1 | *B. longum* subsp. *longum* MCC10062 |
| GCF_004333425.1 | *B. longum* subsp. *longum* MCC10064 |
| GCF_004333385.1 | *B. longum* subsp. *longum* MCC10066 |
| GCF_004333375.1 | *B. longum* subsp. *longum* MCC10067 |
| GCF_004333365.1 | *B. longum* subsp. *longum* MCC10068 |
| GCF_004333335.1 | *B. longum* subsp. *longum* MCC10069 |
| GCF_004333325.1 | *B. longum* subsp. *longum* MCC10070 |
| GCF_004333305.1 | *B. longum* subsp. *longum* MCC10071 |
| GCF_004334205.1 | *B. longum* subsp. *longum* MCC10072 |
| GCF_004333275.1 | *B. longum* subsp. *longum* MCC10073 |
| GCF_004334165.1 | *B. longum* subsp. *longum* MCC10074 |
| GCF_004333265.1 | *B. longum* subsp. *longum* MCC10075 |
| GCF_004333235.1 | *B. longum* subsp. *longum* MCC10076 |
| GCF_004334155.1 | *B. longum* subsp. *longum* MCC10077 |
| GCF_004333205.1 | *B. longum* subsp. *longum* MCC10078 |
| GCF_004333215.1 | *B. longum* subsp. *longum* MCC10079 |
| GCF_004333175.1 | *B. longum* subsp. *longum* MCC10080 |
| GCF_004333165.1 | *B. longum* subsp. *longum* MCC10081 |
| GCF_004334145.1 | *B. longum* subsp. *longum* MCC10083 |
| GCF_004333125.1 | *B. longum* subsp. *longum* MCC10084 |
| GCF_004334105.1 | *B. longum* subsp. *longum* MCC10085 |
| GCF_004333115.1 | *B. longum* subsp. *longum* MCC10086 |
| GCF_004333105.1 | *B. longum* subsp. *longum* MCC10087 |
| GCF_004333065.1 | *B. longum* subsp. *longum* MCC10089 |
| GCF_004333035.1 | *B. longum* subsp. *longum* MCC10090 |
| GCF_004333015.1 | *B. longum* subsp. *longum* MCC10091 |
| GCF_004332925.1 | *B. longum* subsp. *longum* MCC10092 |
| GCF_004332865.1 | *B. longum* subsp. *longum* MCC10093 |
| GCF_004332895.1 | *B. longum* subsp. *longum* MCC10094 |
| GCF_004332855.1 | *B. longum* subsp. *longum* MCC10095 |
| GCF_004333045.1 | *B. longum* subsp. *longum* MCC10096 |
| GCF_004332825.1 | *B. longum* subsp. *longum* MCC10097 |
| GCF_004332835.1 | *B. longum* subsp. *longum* MCC10098 |
| GCF_004332745.1 | *B. longum* subsp. *longum* MCC10099 |
| GCF_004332965.1 | *B. longum* subsp. *longum* MCC10100 |
| GCF_004332725.1 | *B. longum* subsp. *longum* MCC10101 |
| GCF_004332755.1 | *B. longum* subsp. *longum* MCC10102 |
| GCF_004333005.1 | *B. longum* subsp. *longum* MCC10103 |
| GCF_004332735.1 | *B. longum* subsp. *longum* MCC10106 |
| GCF_004332765.1 | *B. longum* subsp. *longum* MCC10107 |
| GCF_004332665.1 | *B. longum* subsp. *longum* MCC10108 |
| GCF_004332655.1 | *B. longum* subsp. *longum* MCC10111 |
| GCF_004332935.1 | *B. longum* subsp. *longum* MCC10112 |
| GCF_004332635.1 | *B. longum* subsp. *longum* MCC10113 |
| GCF_004332625.1 | *B. longum* subsp. *longum* MCC10114 |
| GCF_004332645.1 | *B. longum* subsp. *longum* MCC10115 |
| GCF_004332945.1 | *B. longum* subsp. *longum* MCC10116 |
| GCF_004334745.1 | *B. longum* subsp. *longum* MCC10117 |
| GCF_004334705.1 | *B. longum* subsp. *longum* MCC10118 |
| GCF_004334865.1 | *B. longum* subsp. *longum* MCC10119 |
| GCF_004334715.1 | *B. longum* subsp. *longum* MCC10120 |
| GCF_004334695.1 | *B. longum* subsp. *longum* MCC10121 |
| GCF_004334855.1 | *B. longum* subsp. *longum* MCC10122 |
| GCF_004334815.1 | *B. longum* subsp. *longum* MCC10123 |
| GCF_004334645.1 | *B. longum* subsp. *longum* MCC10124 |
| GCF_004334795.1 | *B. longum* subsp. *longum* MCC10125 |
| GCF_004334635.1 | *B. longum* subsp. *longum* MCC10126 |
| GCF_004334615.1 | *B. longum* subsp. *longum* MCC10127 |
| GCF_004334785.1 | *B. longum* subsp. *longum* MCC10128 |
| GCF_004334775.1 | *B. longum* subsp. *longum* MCC10129 |
| GCF_004334625.1 | *B. longum* subsp. *longum* MCC10130 |
| GCF_004334555.1 | *B. longum* subsp. *longum* MCC10212 |
| GCF_001446255.1 | *B. longum* subsp. *longum* NCIMB8809 |
| GCF_003990235.1 | *B. longum* subsp. *longum* VKPM Ac-1636 |
| GCF_000786175.1 | *B. longum* subsp. *longum* VMKB44 |
| GCF_001940535.1 | *B. longum* subsp. *longum* W11 |
| GCF_005844335.1 | *B. pseudocatenulatum* 1001271st1_F3 |
| GCF_003952825.1 | *B. pseudocatenulatum* 12 |
| GCF_002075945.1 | *B. pseudocatenulatum* 1896B |
| GCF_002271255.1 | *B. pseudocatenulatum* 1E |
| GCF_001405035.1 | *B. pseudocatenulatum* 2789STDY5834840 |
| GCF_003466105.1 | *B. pseudocatenulatum* AF02-36-1 |
| GCF_003465775.1 | *B. pseudocatenulatum* AF03-28 |
| GCF_003465135.1 | *B. pseudocatenulatum* AF11-18 |
| GCF_003465065.1 | *B. pseudocatenulatum* AF12-10-6.0 |
| GCF_003465385.1 | *B. pseudocatenulatum* AF12-8A-LB |
| GCF_003464925.1 | *B. pseudocatenulatum* AF12-8LB-d |
| GCF_003460425.1 | *B. pseudocatenulatum* AF17-20AC |
| GCF_003459865.1 | *B. pseudocatenulatum* AF18-2AC |
| GCF_003459475.1 | *B. pseudocatenulatum* AF20-20AC |
| GCF_003458965.1 | *B. pseudocatenulatum* AF26-1 |
| GCF_003474835.1 | *B. pseudocatenulatum* AF36-12AT |
| GCF_003474525.1 | *B. pseudocatenulatum* AF41-3MH |
| GCF_003474015.1 | *B. pseudocatenulatum* AF45-10BH |
| GCF_003472725.1 | *B. pseudocatenulatum* AM08-2 |
| GCF_003472685.1 | *B. pseudocatenulatum* AM08-25 |
| GCF_003472575.1 | *B. pseudocatenulatum* AM10-2 |
| GCF_003472545.1 | *B. pseudocatenulatum* AM10-24 |
| GCF_003472415.1 | *B. pseudocatenulatum* AM11-10 |
| GCF_003473115.1 | *B. pseudocatenulatum* AM13-2 |
| GCF_003473025.1 | *B. pseudocatenulatum* AM13-8 |
| GCF_003471505.1 | *B. pseudocatenulatum* AM18-42 |
| GCF_003470545.1 | *B. pseudocatenulatum* AM19-19 |
| GCF_003471415.1 | *B. pseudocatenulatum* AM20-1 |
| GCF_003471325.1 | *B. pseudocatenulatum* AM20-6 |
| GCF_003471295.1 | *B. pseudocatenulatum* AM20-9-6.0 |
| GCF_003470125.1 | *B. pseudocatenulatum* AM26-14LB |
| GCF_003468555.1 | *B. pseudocatenulatum* AM33-6 |
| GCF_003467785.1 | *B. pseudocatenulatum* AM36-2AC |
| GCF_003467755.1 | *B. pseudocatenulatum* AM36-5BH |
| GCF_003467515.1 | *B. pseudocatenulatum* AM38-8 |
| GCF_003467065.1 | *B. pseudocatenulatum* AM43-10 |
| GCF_004167565.1 | *B. pseudocatenulatum* ca_0067 |
| GCF_001685965.1 | *B. pseudocatenulatum* CA-05 |
| GCF_001685985.1 | *B. pseudocatenulatum* CA-B29 |
| GCF_001686005.1 | *B. pseudocatenulatum* CA-C29 |
| GCF_001686025.1 | *B. pseudocatenulatum* CA-D29 |
| GCF_001686045.1 | *B. pseudocatenulatum* CA-K29a |
| GCF_001686065.1 | *B. pseudocatenulatum* CA-K29b |
| GCF_000940535.1 | *B. pseudocatenulatum* CECT 7765 |
| GCF_000708005.1 | *B. pseudocatenulatum* IPLA36007 |
| GCF_001025215.1 | *B. pseudocatenulatum* JCM 1200 |
| GCF_003463505.1 | *B. pseudocatenulatum* OF01-12 |
| GCF_003463455.1 | *B. pseudocatenulatum* OF01-2 |
| GCF_003463265.1 | *B. pseudocatenulatum* OF01-21AC |
| GCF_003463425.1 | *B. pseudocatenulatum* OF01-8 |
| GCF_003439655.1 | *B. pseudocatenulatum* OF05-12 |
| GCF_003438405.1 | *B. pseudocatenulatum* OM05-2 |
| GCF_003438015.1 | *B. pseudocatenulatum* OM10-8 |
| GCF_003437835.1 | *B. pseudocatenulatum* TF05-19AC |
| GCF_003437825.1 | *B. pseudocatenulatum* TF05-2AC |
| GCF_003437155.1 | *B. pseudocatenulatum* TF07-23 |
| GCF_003437075.1 | *B. pseudocatenulatum* TF07-45 |
| GCF_003436955.1 | *B. pseudocatenulatum* TF08-3AT |
| GCF_003436675.1 | *B. pseudocatenulatum* TM01-4 |
| GCF_003436545.1 | *B. pseudocatenulatum* TM04-13 |
| GCF_003437435.1 | *B. pseudocatenulatum* TM05-11 |
| GCF_003436315.1 | *B. pseudocatenulatum* TM07-3AT |
| GCF_003436105.1 | *B. pseudocatenulatum* TM08-2 |
| GCF_003436025.1 | *B. pseudocatenulatum* TM10-1 |
| GCF_004155425.1 | *B. pseudolongum* subsp. *globosum* 102015 |
| GCF_004155405.1 | *B. pseudolongum* subsp. *globosum* 102017 |
| GCF_004155855.1 | *B. pseudolongum* subsp. *globosum* 112206 |
| GCF_004155235.1 | *B. pseudolongum* subsp. *globosum* 1511B |
| GCF_002846775.1 | *B. pseudolongum* subsp. *globosum* 1520B |
| GCF_002846815.1 | *B. pseudolongum* subsp. *globosum* 1524B |
| GCF_004155845.1 | *B. pseudolongum* subsp. *globosum* 1546B |
| GCF_002846715.1 | *B. pseudolongum* subsp. *globosum* 1549B |
| GCF_004155805.1 | *B. pseudolongum* subsp. *globosum* 1550B |
| GCF_004155795.1 | *B. pseudolongum* subsp. *globosum* 1565B |
| GCF_004156215.1 | *B. pseudolongum* subsp. *globosum* 1577B |
| GCF_004155745.1 | *B. pseudolongum* subsp. *globosum* 1578B |
| GCF_004156145.1 | *B. pseudolongum* subsp. *globosum* 1616B |
| GCF_002846685.1 | *B. pseudolongum* subsp. *globosum* 1619B |
| GCF_004156195.1 | *B. pseudolongum* subsp. *globosum* 1655B |
| GCF_004155155.1 | *B. pseudolongum* subsp. *globosum* 1678B |
| GCF_002846875.1 | *B. pseudolongum* subsp. *globosum* 1691B |
| GCF_002846845.1 | *B. pseudolongum* subsp. *globosum* 1734B |
| GCF_002846675.1 | *B. pseudolongum* subsp. *globosum* 1744B |
| GCF_002846835.1 | *B. pseudolongum* subsp. *globosum* 1747B |
| GCF_004155705.1 | *B. pseudolongum* subsp. *globosum* 1770B |
| GCF_004168525.1 | *B. pseudolongum* subsp. *globosum* 1780B |
| GCF_004155695.1 | *B. pseudolongum* subsp. *globosum* 1791B |
| GCF_004155375.1 | *B. pseudolongum* subsp. *globosum* 1805B |
| GCF_004155285.1 | *B. pseudolongum* subsp. *globosum* 2000B |
| GCF_004155645.1 | *B. pseudolongum* subsp. *globosum* 2001B |
| GCF_004156235.1 | *B. pseudolongum* subsp. *globosum* 2002B |
| GCF_004155305.1 | *B. pseudolongum* subsp. *globosum* 2003B |
| GCF_004155115.1 | *B. pseudolongum* subsp. *globosum* 2004B |
| GCF_004155625.1 | *B. pseudolongum* subsp. *globosum* 2009B |
| GCF_004155145.1 | *B. pseudolongum* subsp. *globosum* 2012B |
| GCF_004155275.1 | *B. pseudolongum* subsp. *globosum* 2017B |
| GCF_004155095.1 | *B. pseudolongum* subsp. *globosum* 2019B |
| GCF_004155635.1 | *B. pseudolongum* subsp. *globosum* 2023B |
| GCF_004155165.1 | *B. pseudolongum* subsp. *globosum* 2029B |
| GCF_004155615.1 | *B. pseudolongum* subsp. *globosum* 2032B |
| GCF_004155595.1 | *B. pseudolongum* subsp. *globosum* 2048B |
| GCF_004156115.1 | *B. pseudolongum* subsp. *globosum* 2049B |
| GCF_004155135.1 | *B. pseudolongum* subsp. *globosum* 2071B |
| GCF_004155565.1 | *B. pseudolongum* subsp. *globosum* 2072B |
| GCF_004155045.1 | *B. pseudolongum* subsp. *globosum* 2083B |
| GCF_004156135.1 | *B. pseudolongum* subsp. *globosum* 2086B |
| GCF_004156075.1 | *B. pseudolongum* subsp. *globosum* 2088B |
| GCF_004155525.1 | *B. pseudolongum* subsp. *globosum* 2089B |
| GCF_004155535.1 | *B. pseudolongum* subsp. *globosum* 2093B |
| GCF_004155495.1 | *B. pseudolongum* subsp. *globosum* 2098B |
| GCF_004156175.1 | *B. pseudolongum* subsp. *globosum* 2103B |
| GCF_004155505.1 | *B. pseudolongum* subsp. *globosum* 2105B |
| GCF_004155475.1 | *B. pseudolongum* subsp. *globosum* 2109B |
| GCF_004155295.1 | *B. pseudolongum* subsp. *globosum* 2113B |
| GCF_004154995.1 | *B. pseudolongum* subsp. *globosum* 2114B |
| GCF_004156085.1 | *B. pseudolongum* subsp. *globosum* 2115B |
| GCF_004155325.1 | *B. pseudolongum* subsp. *globosum* 22506 |
| GCF_004155435.1 | *B. pseudolongum* subsp. *globosum* 22511 |
| GCF_002706665.1 | *B. pseudolongum* subsp. *globosum* DSM 20092 |
| GCF_002846755.1 | *B. pseudolongum* subsp. *pseudolongum* 1370B |
| GCF_004155835.1 | *B. pseudolongum* subsp. *pseudolongum* 1513B |
| GCF_002846725.1 | *B. pseudolongum* subsp. *pseudolongum* 1595B |
| GCF_004155395.1 | *B. pseudolongum* subsp. *pseudolongum* 1604B |
| GCF_004155715.1 | *B. pseudolongum* subsp. *pseudolongum* 1612B |
| GCF_004155725.1 | *B. pseudolongum* subsp. *pseudolongum* 1629B |
| GCF_004155015.1 | *B. pseudolongum* subsp. *pseudolongum* 2054B |
| GCF_000771225.1 | *B. pseudolongum* subsp. *pseudolongum* DSM 20099 |
| GCF_002846785.1 | *B. thermophilum* 1542B |
| GCF_002846655.1 | *B. thermophilum* 1543B |
| GCF_000771265.1 | *B. thermophilum* DSM 20210 |
| GCF_000687575.1 | *B. thermophilum* DSM 20212 |
| GCF_000347695.1 | *B. thermophilum* RBL67 |
